# Associations of coffee, alcohol, medication and supplement use with the metabolome and lipidome: an observational study of premenopausal women

**DOI:** 10.1007/s11306-026-02467-9

**Published:** 2026-05-24

**Authors:** Saira Khan, Yueshui Lyu, Vaishnavi Mamillapalle, Myung Sik Jeon, Ghazaleh Pourali, Chongliang Luo, Jingqin Luo, Adetunji T. Toriola

**Affiliations:** 1 Department of Surgery, Division of Public Health Sciences, Washington University School of Medicine, 660 South Euclid Avenue Campus Box 8100, St. Louis, MO 63110 USA; 2 Siteman Cancer Center, Washington University School of Medicine, St. Louis, MO USA

**Keywords:** Metabolomics, Lipidomics, Women, Alcohol, Coffee, Supplements, Medications

## Abstract

**Introduction:**

Lifestyle factors have been consistently linked to various health outcomes. However, few studies have comprehensively assessed how multiple of these factors are associated with metabolome and lipidome in a single study.

**Objectives:**

We leveraged untargeted metabolomics and lipidomics to determine the associations of frequencies of alcohol and coffee intake, supplement and medication use, with metabolites and lipid species.

**Methods:**

This study consists of 702 premenopausal women who provided fasting blood samples. Final analysis included 857 metabolites and 828 lipid species. Multivariable linear regression models adjusted for confounders were used to investigate the associations. We corrected for multiple testing using Benjamini–Hochberg false discovery rate (FDR adjusted p-value < 0.01) and Bonferroni correction (Bonferroni-adjusted p-value < 10^− 5^).

**Results:**

At FDR p-value < 0.01, the frequency of alcohol intake was associated with the largest number of biomarkers, including 89 lipid species (9 after Bonferroni correction) across several pathways (ceramides, phospatidylcholines, triacylglycerols, and androgenic steroids) and 123 (13 after Bonferroni correction) metabolites. Frequency of coffee intake was associated with 46 metabolites (27 after Bonferroni correction), with the strongest associations observed for quinate, 3-hydroxypyridine sulfate and trigonelline (N′-methylnicotinate). Frequency of multivitamin use was associated with 18 metabolites (6 after Bonferroni correction). Frequency of statin use was associated with 35 lipid species, respectively, but these were not significant after Bonferroni correction.

**Conclusions:**

Alcohol, coffee, and multivitamins are associated with several metabolites and lipid species. These findings offer valuable insights into potential biomarkers of exposure and molecular pathways through which these exposures may be associated with health outcomes.

**Supplementary Information:**

The online version contains supplementary material available at 10.1007/s11306-026-02467-9.

## Introduction

Lifestyle factors have been consistently linked to various health outcomes, including cancer, cardiovascular disease, and metabolic disorders (Zeng et al. 2022, Veronese et al. 2020, Taylor et al. 2013, Schjerning et al. 2020, Rumgay et al. 2021, Roerecke 2021, Poole et al. 2017, Liu et al. 2022, Li et al. 2018, Kolawole and Kashfi 2022, Khan et al. 2019, Jiang et al. 2021, Harvie 2014, Giustina et al. 2024, Elwood et al. 2022, Cormick et al. 2021, Chou et al. 2022, Bindu et al. 2020). Despite their health impact, data is only just emerging on their broad association with the metabolome and lipidome. Metabolomics and lipidomics offer powerful analytical approaches to identify responses to environmental exposures, enabling the profiling of metabolites and lipid species, and providing insights on the metabolic pathways involved in several cellular processes (Astarita et al. 2023). While studies have demonstrated associations of some exposures with metabolites and lipids, most are limited in scope, often focusing on a single exposure or a narrow set of metabolites (Yang et al. 2024, Harada et al. 2016, Du et al. 2020, Langenau et al. 2019, Jaremek et al. 2013, Liu et al. 2022, Favari et al. 2021, Cornelis et al. 2018, He et al. 2021, Zhu et al. 2023, Kaddurah-Daouk et al. 2010, Dennis et al. 2018, Sachse et al. 2012, Mitro et al. 2021).

Alcohol metabolism involves the oxidation of ethanol to acetaldehyde using alcohol dehydrogenase as a catalyst (Lee et al. 2025) (Zakhari 2006) and may be associated with alterations in amino acids, omega-3-fatty acids, steroids, and tocopherols (Yang et al. 2024, Dorgan et al. 2020). Coffee, contains several bioactive compounds, which are associated with both positive and negative health outcomes (Dam et al. 2020) including reduced overall mortality (Emadi and Kamangar 2025). Conversely, coffee may also be associated with increased risk for hypertension and arrhythmias (Emadi and Kamangar 2025). Multivitamins contain multiple vitamins, minerals, and other nutrients and may be associated with enhanced nutritional balance and energy metabolism (Sánchez et al. 2024, Sánchez et al. 2024). Certain medications also impact metabolic pathways, with statins reducing oxidized lipids and lipoproteins (Dennis et al. 2018, Lim et al. 2014, Stancu and Sima 2001) and leading to improved cardiovascular and overall health outcomes (Ghani Khan et al. 2025). Nonsteroidal anti-inflammatory drugs (NSAIDs) impact inflammatory and metabolic pathways (Ghosh 2021, Ghosh et al. 2024). Combined, this evidence suggests a potential association of these exposures on metabolomic profiles, however, much of the evidence to date comes from studies relying on a few biomarkers and metabolites. Studies have reported associations of alcohol, medications, coffee, and dietary factors with metabolites and lipid species, usually in different populations (Dorgan et al. 2020, Garrett et al. 2023, Hang et al. 2020, Stevens et al. 2023, Suhre et al. 2022, Wang et al. 2020). Here, we comprehensively examine how these exposures are associated with the metabolome and lipidome in a defined population of premenopausal women.

The study population was designed to assess determinants and biomarkers of mammographic breast density in premenopausal women undergoing annual screening mammogram (Getz et al. 2023). Specifically, the objective of this study is to assess the associations of, frequency of alcohol intake, frequency of coffee intake, frequency of supplement use (calcium, vitamin D, multivitamin), and frequency of medications use (aspirin, acetaminophen, ibuprofen, and statins) with metabolites and lipid species in women using untargeted metabolomic and lipidomic profiling. This study is one of the first study to examine these exposures together in a defined population of premenopausal women.

## Materials and methods

### Study population

This study included 705 premenopausal women who had their annual screening mammograms at Washington University School of Medicine (WUSM) in St. Louis, MO. Recruitment occurred in two periods between December 2015 - October 2016 and September 2020 - February 2020. Eligibility criteria have been published previously (Getz et al. 2023). Women were included if they were premenopausal and not pregnant at the time of their mammograms. Women were identified as premenopausal if they had a regular menstrual period within the preceding 12 months, no prior history of bilateral oophorectomy, and had not used menopausal hormone therapy. Women were excluded if they had a history of cancer, breast augmentation (implants or reduction), and currently use or have used selective estrogen receptor modulators in the prior six months. Participants completed validated (Evans et al. 2016) questionnaires capturing demographic, behavioral, lifestyle characteristics, and provided fasting blood samples for biomarker analyses on the day of their mammograms. For questions regarding alcohol intake, participants were asked, “How many alcoholic beverages do you typically consume per week?”. Alcohol response categories included: Less than 1 drink per week, 1–2 drinks per week, 3–5 drinks per week, and 6–10 drinks per week, and more than 10 drinks per week. For questions regarding coffee intake participants were asked, “How many servings do you typically consume of the following: Coffee (8 oz glass)?”. Coffee response categories included: Never or less than one per week, 1 per week, 2–6 per week, 1 per day, 2–3 per day, 4–5 per day, and 6 + per day. For questions regarding medication and supplement frequency, participants were asked, “In the past 12 months, how often did you take the following medications/supplements (average number of days per week)?”. Response categories for calcium, vitamin D, and multivitamins included: Never, less than 1 day per week, 1–3 days per week, 4–6 days per week, and everyday. Responses categories for acetaminophen, aspirin, Ibuprofen, and statins included: Never, 1 day, 2–3 days, 4–5 days, and 6 + days.

Plasma was extracted from blood samples and stored at −80℃ within 30 min of collection at WUSM Siteman Cancer Center Tissue Procurement Core, following their established protocols (Toriola et al. 2018). The final analytic cohort comprised 702 participants after excluding 3 women with missing questionnaires information. A flow diagram of included participants is included in Supplementary Figure S1. The study was approved by the Institutional Review Board at Washington University in Saint Louis (#201505122) and conducted in accordance with the Declaration of Helsinki. All participants provided written informed consent.

## Lipidomic and metabolomic profiling

Untargeted lipidomic and metabolomic profiling was performed by Metabolon (Durham, NC, USA) (Metabolon 2023), quantifying 982 lipid species and 1074 metabolites and full details have been provided in our prior studies (Getz et al. 2023, Matthew et al. 2024). We excluded 125 of the 982 lipid species, and 246 of the 1,074 metabolites detected due to excessive missing (in > 300 participants). The remaining missing values were imputed using the 10-nearest neighbor method (Schaibley et al. 2022). Imputations were performed separately for lipids and metabolites using the “impute” R package. Batch effects were mitigated by applying ComBat normalization to peak area metabolite data (Johnson et al. 2007). The use of ComBat for batch normalization is well-accepted in omics data (Wen et al. 2017, Yu et al. 2023).

## Study variables

Frequency of alcohol intake was classified as: never, < 1 drink/week, 1–3 drinks/week, 3–5 drinks/week, and ≥ 6 drinks/week; In addition, a binary frequency of alcohol intake variable was created by collapsing all categories other than never into ever. Frequency of coffee intake was categorized as never, < 1 serving/week, 1 serving/week, 2–6 servings/week, and ≥ 7 servings/week. Supplements included calcium, vitamin D, and multivitamins, each categorized as: never, ≤ 3 days/week, 4–6 days/week, and daily. Medications included aspirin, acetaminophen, ibuprofen, and statins. All were categorized as: never, 1 day/week, 2–3 days/week, and > 4 days/week, except for frequency of statin use, which was categorized as: never, 1–3 days/week, and ≥ 4 days/week, due to small sample sizes in some categories.

### Statistical analysis

We summarized the distribution of characteristics using means and standard deviation (SD) for continuous variables, and percentages for categorical variables. Multivariable linear regression models were used to evaluate the associations of exposures of interest [frequency of alcohol intake, frequency of coffee intake, frequency of supplement use (calcium, vitamin D, multivitamin), and frequency of medication use (aspirin, acetaminophen, ibuprofen, and statins)] on metabolites and lipid species, adjusting for age (continuous), body mass index (BMI, kg/m²), race (non-Hispanic White, non-Hispanic Black, Remaining), education (high school or less, some college, college graduate, postgraduate), and binary alcohol intake (ever, never). Each exposure was analyzed in a separate model. Regression models assessing alcohol as the primary exposure did not include frequency of alcohol intake as a covariate. Least square means (LSMs) of metabolites and lipid species were estimated across categories of each exposure using multivariable regression models. To evaluate dose–response relationships, p-values for trend were obtained using polynomial contrast analysis, with exposure categories modeled as ordinal variables. To improve the precision of interval coding for variables with unequal category ranges, we assigned numeric scores using interval midpoints for closed categories and the lower bound for the open-ended highest category. Metabolites and lipid species were log10 transformed to improve normality. We corrected for multiple testing using both false discovery rate (FDR) correction and Bonferroni correction. We present the more conservative correction (Bonferroni-adjusted p-value < 10^−5^) for primary results visualized in the figures while significant results based on both FDR-adjusted p-value < 0.01 and Bonferroni corrections (Bonferroni-adjusted p-value < 10^−5^) in more detail in the supplementary tables. Analyses were performed using the R statistical software (v 4.5.0).

## Results

Characteristics of the study population are summarized in Table 1. Mean age and BMI were 46 years and 30 kg/m², respectively. The majority were non-Hispanic White (71.7%), and then non-Hispanic Black (23.2%).

**Table 1:** Characteristics of 702 premenopausal women recruited during annual screening mammography^a^

Characteristic	*N*	Mean ± SD/Percentage^b^
Age (years)	702	46.0 ± 4.5
BMI (kg/m^**2**^**)**	702	30.0 ± 7.5
Race		
Non-Hispanic White (NHW)	503	71.7%
Non-Hispanic Black (NHB)	163	23.2%
Other	36	5.1%
Education		
High school and below	53	7.5%
Post high school	108	15.4%
College graduate	244	34.8%
Postgraduate	262	37.3%
Missing	35	5.0%
Frequency of Alcohol Intake		
Never	211	30.1%
<1 Drink	180	25.6%
1–2 Drinks	133	18.9%
3–5 Drinks	126	17.9%
≥6 Drinks	51	7.3%
Missing	1	0.1%
Frequency of Coffee Intake		
Never or < 1 serving^c^	230	32.8%
1 serving	33	4.7%
2–6 servings	72	10.3%
≥7 servings	356	50.7%
Missing	11	1.5%
Frequency of Calcium Use		
Never	506	72.1%
≤3 days	78	11.1%
4–6 days	22	3.1%
Everyday	84	12.0%
Missing	12	1.7%
Frequency of Vitamin D Use		
Never	359	51.1%
≤3 Days	114	16.2%
4–6 Days	37	5.3%
Everyday	180	25.6%
Missing	12	1.7%
Frequency of Multivitamin Use		
Never	320	45.6%
≤3 Days	105	14.9%
4–6 Days	63	8.9%
Everyday	209	29.8%
Missing	5	0.7%
Frequency of Aspirin Use		
Never	595	84.8%
1 Day	51	7.3%
2–3 Days	17	2.4%
≥4 Days	23	3.3%
Missing	16	2.3%
Frequency of Acetaminophen Use		
Never	382	54.4%
1 Day	150	21.4%
2–3 Days	74	10.5%
≥4 Days	83	11.8%
Missing	13	1.8%
Frequency of Ibuprofen Use		
Never	236	33.6%
1 Day	214	30.5%
2–3 Days	130	18.5%
≥4 Days	114	16.2%
Missing	8	1.1%
Frequency of Statins Use^d^		
Never	625	89.0%
1–3 Days	20	2.9%
≥4 Days	37	5.2%
Missing	20	2.9%

^a^Participants were recruited at the Joanne Knight Breast Health Center, Washington University School of Medicine, St. Louis, MO

^b^Continuous variables are presented as mean ± standard deviation; categorical variables are presented as percentages

^c^1 serving = 8 oz glass

^d^Categories for frequency of statin use have been reclassified, combining the 1 day and 2–3 days categories into 1–3 days group due to the small sample size in the 2–3 days category

Alcohol and statins were associated with lipid species while alcohol, coffee, multivitamins, and statins were associated with metabolites after correcting for multiple testing, with the total number of markers depending on the correction threshold (FDR p-value < 0.01 or Bonferroni *p-*value < 10^−5^) (Table 2). Detailed lists of significant lipid species and metabolites, their corresponding sub-pathways, and LSMs across categories, are provided in Supplementary Table S1-S4. Heatmaps of least squares means for significant metabolites and a Sankey plot of significant metabolite sub-pathways across exposures are presented in Supplementary Figures S2 and S3.

**Fig. 1:**
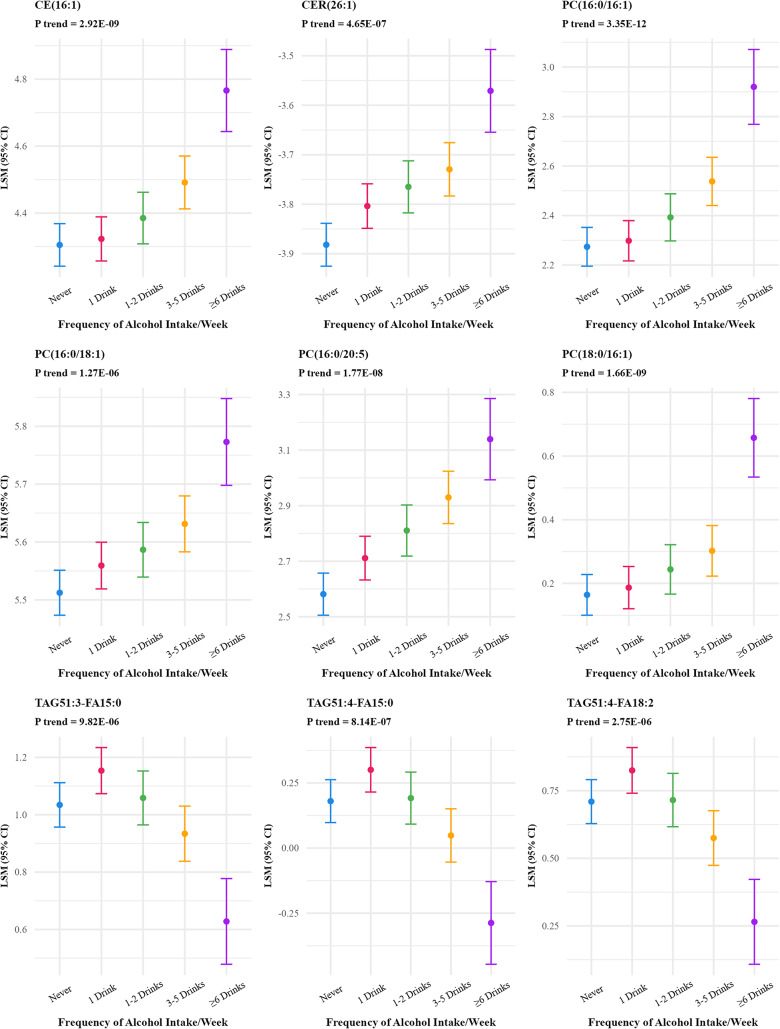
Adjusted least square means of lipid species associated with frequency of alcohol intake at Bonferroni adjusted p value < 10^−5^^abca^Lipid Species were log-transformed.^b^Y-axis scales differ between panels to best display variability within each lipid species.^c^9 out of 857 lipid species were significant at Bonferroni p-value < 10^−5^ threshold and are included in the figure

**Fig. 2:**
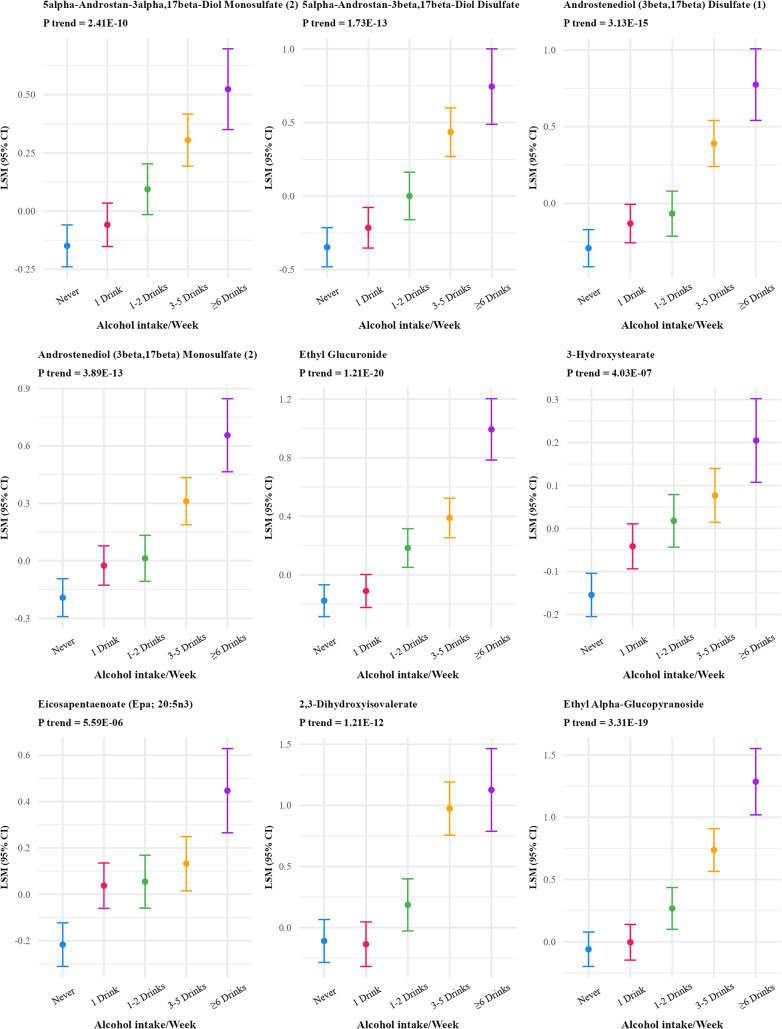

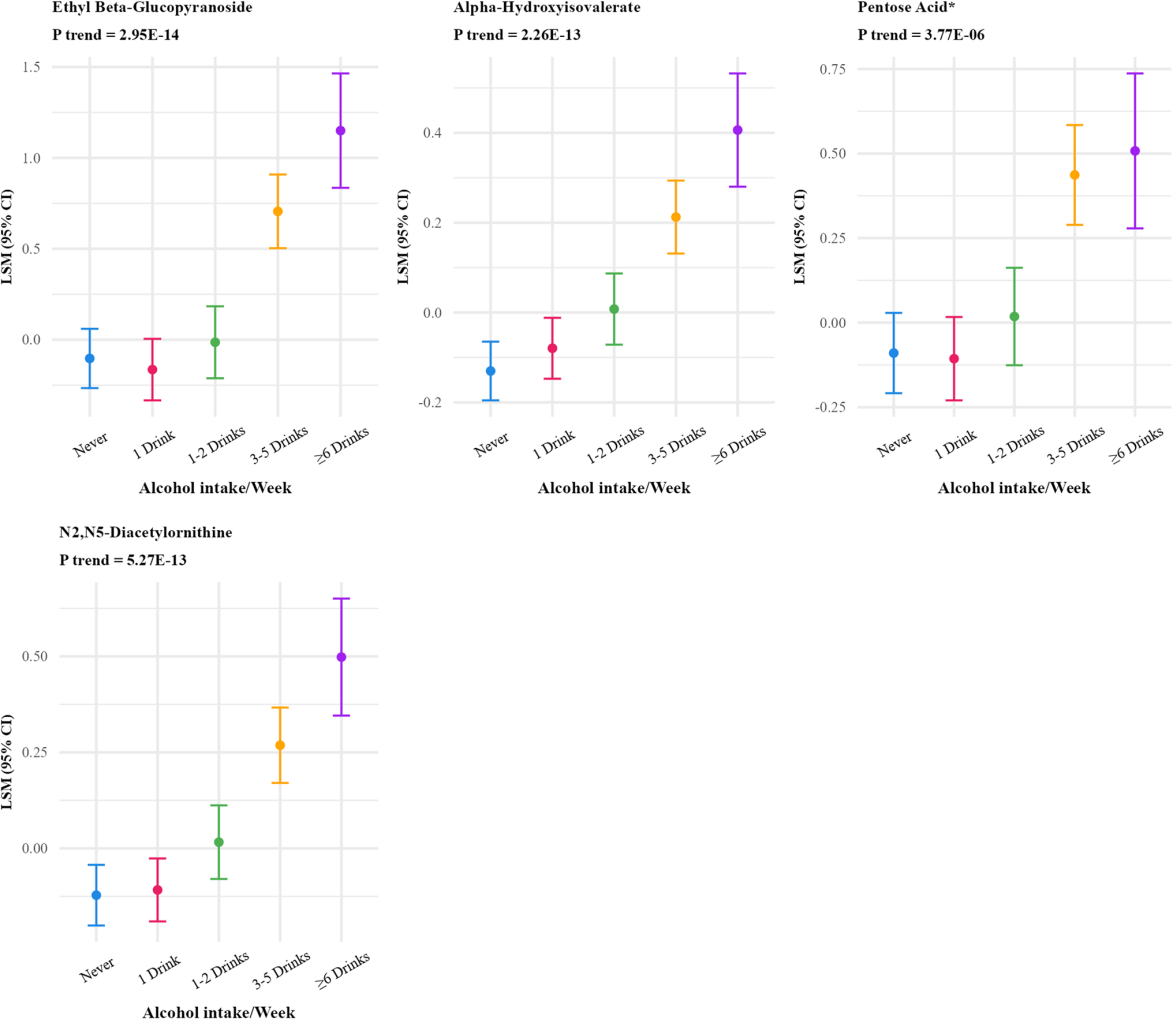
Adjusted least square means of metabolites associated with frequency of alcohol intake at bonferroni adjusted p value < 10^−5^^abc^. ^a^Metabolites were log-transformed.^b^Y-axis scales differ between panels to best display variability within each metabolite.^c^13 out of 828 metabolites were significant at Bonferroni p-value < 10^−5^ threshold and are included in the figure

Frequency of alcohol intake was associated with the greatest number of lipid species and metabolites, 89 lipid species and 123 metabolites at FDR p-value < 0.01 (Supplementary Table S1), with the numbers reducing to 9 lipid species [(CE(16:1), CER(26:1), PC(16:0/16:1), PC(18:0/16:1), PC(16:0/20:5), PC(16:0/18:1), TAG51:4-FA15:0, TAG51:4-FA18:2, and TAG51:3-FA15:0)] and 13 metabolites based on the more stringent Bonferroni correction (p value < 10^−5^) (Table 2; Figs. 1 and 2). The strongest positive associations for metabolites were observed for ethyl glucuronide, ethyl alpha-glucopyranoside, ethyl beta-Glucopyranoside, androstenediol (3beta,17beta) disulfate (1), 5alpha-androstan-3beta,17beta-diol disulfate, and alpha-hydroxyisovalerate.

**Table 2:** Number of lipid species and metabolites associated with the frequency of coffee, alcohol, supplement and medication^ab^

Characteristic	Lipid Species		Metabolite	
	FDR adjusted *p* < 0.01	Bonferroni adjusted *p* < 10^−5^	FDR adjusted *p* < 0.01	Bonferroni adjusted *p* < 10^−5^
Lifestyle				
Frequency of Alcohol Intake	89	9	123	13
Frequency of Coffee Intake	0	0	46	27
Supplement frequency				
Frequency of Calcium Use	0	0	0	0
Frequency of Vitamin D Use	0	0	0	0
Frequency of Multivitamin Use	0	0	18	6
Medication frequency				
Frequency of Aspirin Use	0	0	0	0
Frequency of Acetaminophen Use	0	0	0	0
Frequency of Ibuprofen Use	0	0	0	0
Frequency of Statin Use	35	0	4	0

^a^Multivariable linear regression models were performed on log-transformed lipid species and metabolites. Models were adjusted for age, BMI, education, race, and alcohol intake. Models for frequency of alcohol intake were adjusted for age, BMI, education and race

^b^Associations were considered statistically significant at a false discovery rate (FDR) < 0.01 or a Bonferroni adjusted p value < 10^−5^

**Fig. 3:**
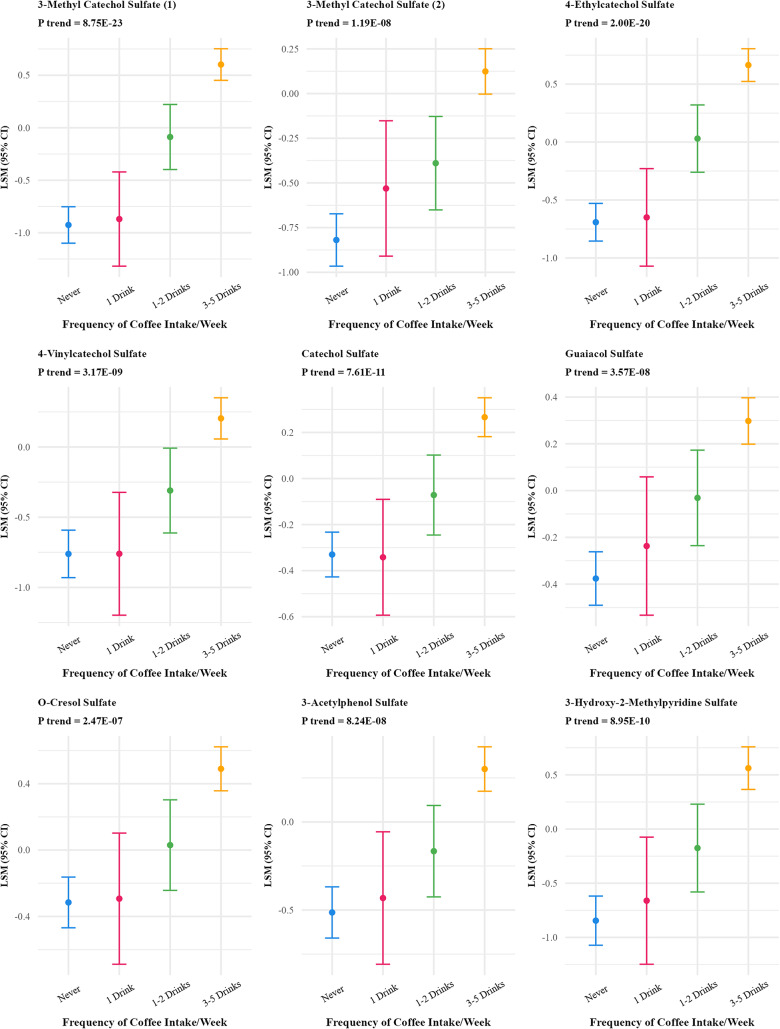

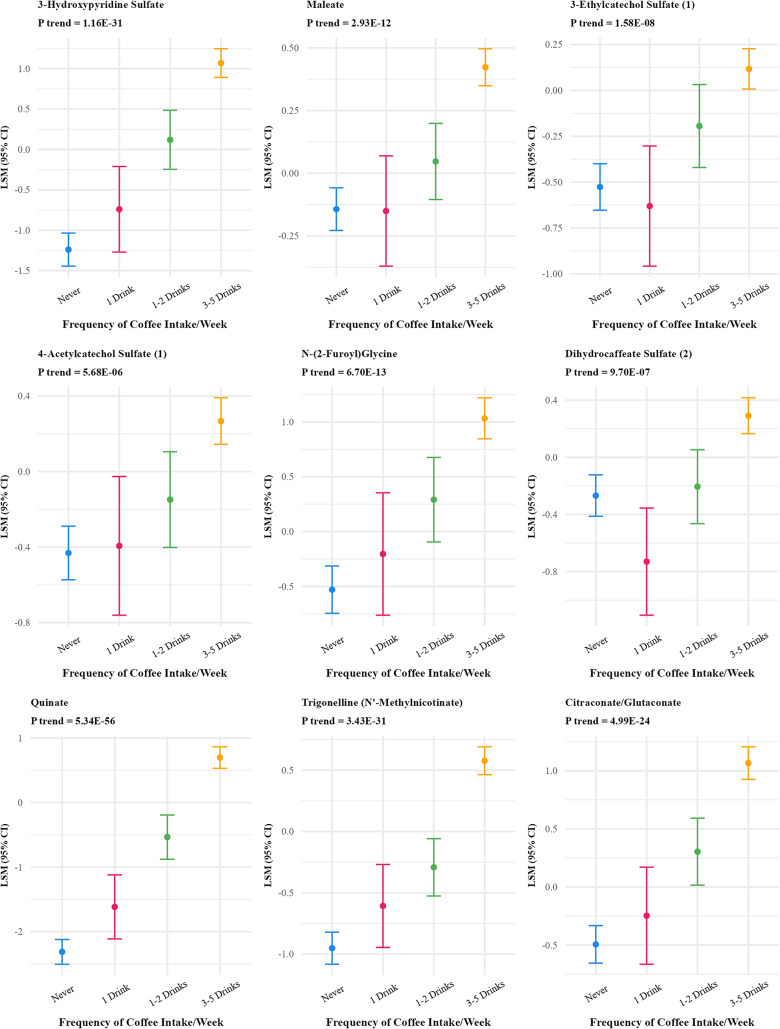

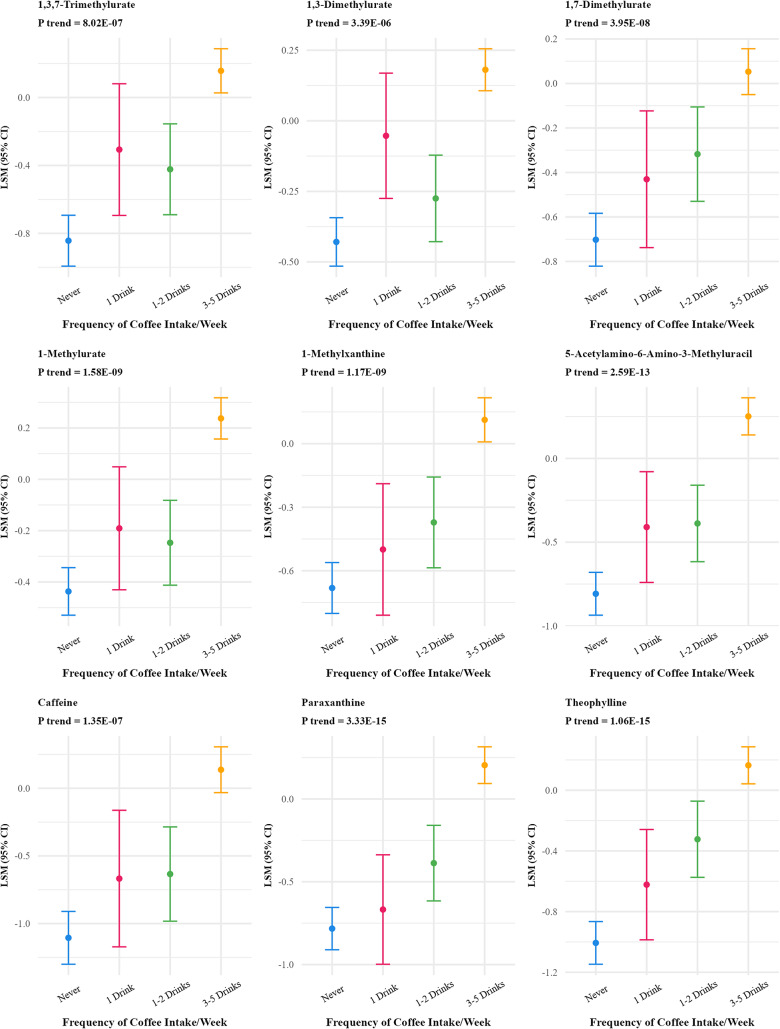
Adjusted least square means of metabolites associated with frequency of coffee intake at bonferroni adjusted p value < 10^−5^^abc^. ^a^Metabolites were log-transformed. ^b^Y-axis scales differ between panels to best display variability within each metabolite. ^c^27 out of 828 metabolites were significant at Bonferroni p-value < 10^−5^ threshold and are included in the figure

**Fig. 4:**
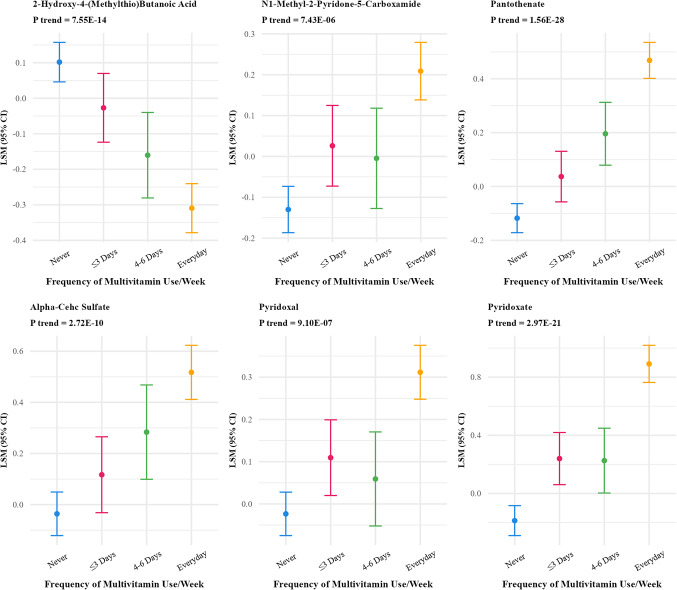
Adjusted least square means of metabolites associated with frequency of multivitamin use at bonferroni adjusted p value < 10^−5^^abc^. ^a^Metabolites were log-transformed.^b^Y-axis scales differ between panels to best display variability within each metabolite.^c^6 out of 828 metabolites were significant at Bonferroni p-value < 10^−5^ threshold and are included in the figure

Frequency of coffee intake was positively associated with 46 metabolites (FDR p-value < 0.01) (Supplementary Table S2) which reduced to 27 after Bonferroni correction (Table 2; Fig. 3). The strongest associations were observed for quinate, 3-hydroxypyridine sulfate, trigonelline (N′-methylnicotinate), citraconate/glutaconate, and 3-methyl catechol sulfate (1). Multivitamin frequency was associated with 18 metabolites (FDR p-value < 0.01) which reduced to 6 after Bonferroni correction (Fig. 4), positively associated with five metabolites (N1-methyl-2-pyridone-5-carboxamide, pantothenate, alpha-CEHC sulfate, pyridoxate and pyridoxal) and inversely associated with one metabolite(2-hydroxy-4-(methylthio) butanoicacid).

Frequency of statin use was associated with 35 lipid species and 4 metabolites at FDR p-value < 0.01, but these were no longer significant after Bonferroni correction (Table 2, Supplementary Table S4). The strongest associations were observed for HCER(24:1), HCER(20:0), HCER(16:0), CE(24:1), HCER(24:0), and HCER(22:0). Positive associations were observed for salicyluric glucuronide* and glucose while an inverse association was observed for 1,5-anhydroglucitol (1,5-AG).

Among lipids identified as significantly associated with individual factors, we summarized features showing pairwise overlapping associations across exposures. Supplementary Tables S6 and S7 report the linear trend coefficients (β) and standard errors (SE) from multivariable models, where ordinal exposures were evaluated using prespecified category scores. Two lipid species, CE(18:3) and CER(24:1), were associated with both frequency of alcohol intake and frequency of statin use (Supplementary Table S6). In both scenarios, there were positive associations with frequency of alcohol intake but inverse associations with frequency of statin use. Likewise, 15 metabolites were positively associated with both frequency of alcohol and coffee intake; 2 metabolites 3-(4-hydroxyphenyl)lactate and cyclo(leu-pro) were positively associated with frequency of alcohol intake and inversely associated with frequency of multivitamin use (Supplementary Table S7). The overlap of significant metabolites across exposures is further illustrated in Supplementary Figure S4 using an UpSet plot.

## Discussion

We investigated the associations of several factors including alcohol, coffee, supplements, and frequently used medications on the lipidome and metabolome in premenopausal women. These findings demonstrate that alcohol and coffee are associated with the most significant changes in the lipidome and metabolome. By contrast, we observed that medications (aspirin, acetaminophen, ibuprofen, and statins) had limited associations with the lipidome and metabolome.

### Alcohol

Consistent with findings from previous studies using targeted and untargeted approaches (Yang et al. 2024, Harada et al. 2016, Du et al. 2020, Langenau et al. 2019, Jaremek et al. 2013, Liu et al. 2022), frequency of alcohol intake was significantly associated with increases in several lipid species and metabolites. Previous studies have reported broad associations for metabolites in lipid, amino acid, carbohydrate, and nucleotide metabolism including phosphatidylcholines (PCs) (Du et al. 2020, Langenau et al. 2019, Jaremek et al. 2013), sphingolipids (Jaremek et al. 2013, Liu et al. 2022), and acylcarnites (Langenau et al. 2019, Liu et al. 2022). In particular, we observed strong associations with PCs, that were consistent with previous findings, ceramides, and novel associations with androgenic steroids. CER 26:1 was the most strongly associated ceramide in our study. CER 26:1 is a long-chain sphingolipid and regulates critical cell functions, most notably apoptosis, differentiation, and inflammatory signaling (Stith et al. 2019). Dysregulation of CER 26:1 may be implicated in metabolic disease and cancer (Wajapeyee et al. 2024). Several phosphatidylcholines were also associated with frequency of alcohol intake, notably PC (16:0/16:1). Phosphatidylcholines are critical for proper functioning of cell membranes (Veen et al. 2017). Alterations in PCs are associated with overall energy metabolism and can be associated with disease progression including alcoholic fatty liver disease (Veen et al. 2017). Several androgenic steroids were strongly associated with alcohol in our study with particularly strong associations observed for androstenediol (3beta,17beta) disulfate, 5alpha-androstan-3beta,17beta-diol disulfate, and androstenediol (3beta,17beta) monosulfate. Previously, in the Dietary Intervention Study in Children 2006 Follow-Up Study (DISC06) (*n* = 211), alcohol was borderline significantly associated with 4-androsten-3beta,17beta-diol disulfate (Dorgan et al. 2020). Our larger study was able to overcome the limitation of sample size and show a significant association for multiple androgenic steroids. Among the 13 metabolic species significantly associated with frequency of alcohol intake, the strongest association was observed for ethyl glucuronide (p-trend: 1.21 × 10^−20^), a highly sensitive and specific biomarker for recent alcohol use that was also identified in other studies (Wang et al. 2022, Wurst et al. 2003).

### Coffee

Over 100 metabolites have previously been associated with coffee (Favari et al. 2021, Cornelis et al. 2018, He et al. 2021). We observed significant frequency-based associations between coffee intake and 27 metabolites, strongest for quinate, 3-hydroxypyridine sulfate, trigonelline (N′-methylnicotinate), citraconate/glutaconate (Bonferroni p-value < 10^−5^). Both quinate and trigonelline are biomarkers of coffee intake (He et al. 2021, Hang et al. 2020). Quinate has antioxidant effects. Trigonelline is a key alkaloid in coffee beans (Iglesias-Carres et al. 2021) and has potential anti-inflammatory, anti-glycemic and anti-oxidative effects and may also protect against apoptosis (Kambalapally et al. 2023). 3-hydroxypyridine sulfate has been associated with bone mineral density (Chau et al. 2020). However, we observed no association between lipid species and frequency of coffee intake.

In a previous randomized control trial, participants in the coffee drinking group experienced increases in cholesteryl esters while oxysterols and free fatty acids decreased (Lara-Guzmán et al. 2021). In another study of habitual coffee drinkers, three lipid species were significantly associated [LPC (20:4), LPC (22:1) and LPC (22:2)] with high coffee intake (four or eight cups of coffee per day) (Kuang et al. 2018). Coffee consumption in our study population was not as high an in the trial, hence, it is possible that the lipidomic changes occur with higher levels of coffee intake, however, it is important to note that we do not have information on the upper bound of coffee consumption since the highest category was open-ended (i.e., > seven servings per week).

### Multivitamins

Our analysis identified six metabolites significantly associated with frequency of multivitamin use. Among these, pantothenate, pyridoxate, alpha-CEHC sulfate, pyridoxal, and N1-methyl-2-pyridone-5-carboxamide demonstrated frequency-based increase across categories. These metabolites are well-established as vitamin-derived compounds and likely reflect the direct contribution of B-complex vitamins and vitamin E from multivitamin formulations. Notably, pantothenate and the vitamin B6 derivatives (pyridoxate and pyridoxal) have been previously associated with supplementation use in other studies, further supporting their role as reliable biomarkers of multivitamin or micronutrient frequency (Sánchez et al. 2024). Interestingly, one metabolite, 2-hydroxy-4-(methylthio)butanoic acid, was inversely associated with frequency of multivitamin use. While this may appear counterintuitive, it may reflect the biological regulation or compensatory feedback mechanisms in nutrient metabolism, or possible differences in the bioavailability or absorption of metabolites in individuals using supplements versus those not using supplements. Previous studies have shown that while water-soluble vitamin metabolites tend to increase with supplementation, lipid-soluble compounds such as tocopherols may have more variable responses depending on formulation, metabolism, and dietary interactions (Sullivan et al. 2025). We did not observe any significant associations with individual supplements including vitamin D and calcium. These findings must be interpreted with caution given the variety of multivitamins available with different compositions, limited information on dose and duration of use, and other factors including physical activity, body compensation, and diet that could have confounded our findings.

### Medications

Among the medications examined (aspirin, acetaminophen, ibuprofen, and stains), statins were the only medications in our study associated with lipid species, being inversely associated with 35 lipid species, using the more relaxed FDR p-value, but none with the stricter Bonferroni correction. This could be due to the low frequency of statin use in the study population, with 89% never having used a statin. Interestingly, we observed a positive association with glucose (using FDR p-value), in support of previous findings that indicate that statins can contribute to insulin resistance (Lim et al. 2014). Statins reduce cholesterol by inhibiting 3-hydroxy-3methylglutaryl-coenzyme A (HMG-CoA) reductase and stimulating the expression of LDL cholesterol receptors (Lim et al. 2014, Stancu and Sima 2001, Vaughan and Gotto 2004). Importantly, the observed association between cholesterol and the lipidome can vary based on “good” vs. “poor” responders to statins. Response to statins can vary due to medication adherence, underlying genetics, and other factors (Trompet et al. 2016).

We observed no associations between aspirin, ibuprofen, or acetaminophen with either metabolites or lipid species. A previous study of older individuals (average age of women participants was 68) found that metabolites are a useful marker to validate self-reported acetaminophen use, but a poor marker of ibuprofen use (Dennis et al. 2018). However, despite this NSAIDs can potentially impact key metabolic pathways (Ghosh 2021). The lack of significant findings in our study could reflect lower and inconsistent use of these medications and the limitations of self-report.

### Strengths and limitations

This study presents a comprehensive investigation into the associations of frequency of alcohol intake, frequency of coffee intake, frequency of supplement use (calcium, vitamin D, multivitamin), and frequency of medications use (aspirin, acetaminophen, ibuprofen, and statins) with the lipidome and metabolome. A major strength lies in the metabolomic and lipidomic profiling of a relatively large sample of premenopausal women in a well-defined population, paired with rigorous corrections for multiple testing. The granularity of metabolites and lipid species profiled, supported by biologically plausible pathways, adds depth to the findings.

Nonetheless, several limitations are acknowledged. Due to the cross-sectional nature of this study, causal inferences cannot be drawn, and temporal relationships between exposures and outcomes cannot be evaluated. Additionally, cholesterol-lowering medications are more commonly prescribed to older adults; however, our study focused exclusively on premenopausal women, which led to a limited number of relevant observations and may restrict the generalizability of the findings to broader age groups. We did not have information on cardiovascular and metabolic disease status, and other lifestyle factors including sleep, diet, and stress that may have potentially confounded our findings. Furthermore, exposures may be subject to misclassification, as they were derived from self-reported weekly estimates rather than precise quantitative measures. Moreover, we did not have detailed information on the time frame, dose, and duration of exposures. Finally, the metabolic response to alcohol, coffee and medications can vary across individuals due to underlying genetics factors, and we are unable to distinguish slow metabolizers from fast metabolizers. Despite these limitations, we did report several significant associations, particularly for frequency of alcohol and coffee intake. Future research should aim to include a wider age range, incorporating postmenopausal women and men to capture potential sex- and age-related metabolic variations. Longitudinal study designs are also recommended to better establish temporal sequences and clarify potential causal mechanisms.

## Conclusions

Alcohol, coffee, and multivitamins are associated with several metabolites and lipid species. These findings provide robust evidence of these associations in a defined population of premenopausal women.

## Supplementary Information

Below is the link to the electronic supplementary material.

Supplementary file1 (DOCX 1703 KB)

Supplementary file1 (DOCX 4156 KB)

## Data Availability

The data generated in this study are available upon request from the corresponding author.

## References

[CR1] Astarita, G., Kelly, R. S., & Lasky-Su, J. (2023). Metabolomics and lipidomics strategies in modern drug discovery and development. *Drug Discovery Today,**28*, Article 103751. 37640150 10.1016/j.drudis.2023.103751 PMC10543515

[CR2] Bindu, S., Mazumder, S., & Bandyopadhyay, U. (2020). Non-steroidal anti-inflammatory drugs (NSAIDs) and organ damage: A current perspective. *Biochemical Pharmacology,**180*, Article 114147. 32653589 10.1016/j.bcp.2020.114147 PMC7347500

[CR3] Chau, Y. P., Au, P. C. M., Li, G. H. Y. (2020). Serum Metabolome of Coffee Consumption and its Association With Bone Mineral Density: The Hong Kong Osteoporosis Study. *The Journal of clinical endocrinology and metabolism*. 105. 10.1210/clinem/dgz210 31750515

[CR4] Chou, R., Cantor, A., Dana, T., et al. (2022). Statin Use for the Primary Prevention of Cardiovascular Disease in Adults: Updated Evidence Report and Systematic Review for the US Preventive Services Task Force. *Jama*, *328*, 754–771. 35997724 10.1001/jama.2022.12138

[CR5] Cormick, G., Betran, A. P., Romero, I. B., et al. (2021). Effect of Calcium fortified foods on health outcomes: A systematic review and meta-analysis. *Nutrients*. 10.3390/nu13020316 33499250 10.3390/nu13020316 PMC7911363

[CR6] Cornelis, M. C., Erlund, I., Michelotti, G. A., Herder, C., Westerhuis, J. A., & Tuomilehto, J. (2018). Metabolomic response to coffee consumption: Application to a three-stage clinical trial. *Journal of Internal Medicine,**283*, 544–557. 29381822 10.1111/joim.12737

[CR7] Dennis, K. K., Carter, B. D., Gapstur, S. M., & Stevens, V. L. (2018). Metabolomics approach for validation of self-reported ibuprofen and acetaminophen use. *Metabolites,**8*, 55. 30248901 10.3390/metabo8040055 PMC6316588

[CR8] Dorgan, J. F., Jung, S., Dallal, C. M., et al. (2020). Alcohol consumption and serum metabolite concentrations in young women. *Cancer Causes & Control: CCC,**31*, 113–26. 31828464 10.1007/s10552-019-01256-1 PMC7008965

[CR9] Du, D., Bruno, R., Blizzard, L., et al. (2020). The metabolomic signatures of alcohol consumption in young adults. *European Journal of Preventive Cardiology*, *27*, 840–849. 30857428 10.1177/2047487319834767

[CR10] Elwood, P., Protty, M., Morgan, G., Pickering, J., Delon, C., & Watkins, J. (2022). Aspirin and cancer: Biological mechanisms and clinical outcomes. *Open Biology,**12*, Article 220124. 36099932 10.1098/rsob.220124 PMC9470249

[CR11] Emadi, R. C., & Kamangar, F. (2025). Coffee’s Impact on Health and Well-Being. Nutrients. 17. 10.3390/nu17152558 PMC12348139 40806142

[CR12] Evans, D. G. R., Donnelly, L. S., Harkness, E. F., et al. (2016). Breast cancer risk feedback to women in the UK NHS breast screening population. *British Journal of Cancer*, *114*, 1045–1052. 27022688 10.1038/bjc.2016.56 PMC4984905

[CR13] Favari, C., Righetti, L., Tassotti, M., et al. (2021). Metabolomic Changes after Coffee Consumption: New Paths on the Block. *Molecular nutrition & food research*, *65*, e2000875. 33300301 10.1002/mnfr.202000875

[CR14] Garrett, T. J., Puchowicz, M. A., Park, E. A., et al. (2023). Effect of statin treatment on metabolites, lipids and prostanoids in patients with Statin Associated Muscle Symptoms (SAMS). *PLoS One,**18*, Article e0294498. 38100464 10.1371/journal.pone.0294498 PMC10723679

[CR15] Getz, K. R., Jeon, M. S., Luo, C., Luo, J., & Toriola, A. T. (2023). Lipidome of mammographic breast density in premenopausal women. *Breast cancer research: BCR*, *25*, 121. 37814330 10.1186/s13058-023-01725-1 PMC10561435

[CR16] Ghani Khan, K., Kaur, P., Bhagat, M., et al. (2025). Effect of Statin Therapy on Clinical Outcomes in Patients With Cardiovascular Risks: A Systematic Review and Meta-Analysis. *Cureus*, *17*, e88238. 40831859 10.7759/cureus.88238 PMC12359277

[CR17] Ghosh, S. (2021). Metabolomic Studies for Metabolic Alterations Induced by Non-Steroidal Anti-Inflammatory Drugs: Mini Review. Biomolecules. 11. 10.3390/biom11101456 PMC8533408 34680089

[CR18] Ghosh, S., Lahens, N., Barekat, K., et al. (2024). *Metabolomic Response to Non-Steroidal Anti-Inflammatory Drugs*. bioRxiv.

[CR19] Giustina, A., Bilezikian, J. P., Adler, R. A., et al. (2024). Consensus Statement on Vitamin D Status Assessment and Supplementation: Whys, Whens, and Hows. *Endocrine reviews*, *45*, 625–654. 38676447 10.1210/endrev/bnae009 PMC11405507

[CR20] Hang, D., Zeleznik, O. A., He, X., et al. (2020). Metabolomic signatures of long-term coffee consumption and risk of type 2 diabetes in women. *Diabetes Care,**43*, 2588–96. 32788283 10.2337/dc20-0800 PMC7510042

[CR21] Harada, S., Takebayashi, T., Kurihara, A., et al. (2016). Metabolomic profiling reveals novel biomarkers of alcohol intake and alcohol-induced liver injury in community-dwelling men. *Environmental health and preventive medicine*, *21*, 18–26. 26459263 10.1007/s12199-015-0494-y PMC4693765

[CR22] Harvie, M. (2014). Nutritional supplements and cancer: potential benefits and proven harms. American Society of Clinical Oncology educational book. American Society of Clinical Oncology. Annual Meeting. e478-86. 10.14694/EdBook_AM.2014.34.e478 24857143

[CR23] He, W. J., Chen, J., Razavi, A. C., et al. (2021). Metabolites Associated with Coffee Consumption and Incident Chronic Kidney Disease. *Clinical journal of the American Society of Nephrology: CJASN*, *16*, 1620–1629. 34737201 10.2215/CJN.05520421 PMC8729408

[CR24] Iglesias-Carres, L., Hughes, M. D., Steele, C. N., Ponder, M. A., Davy, K. P., & Neilson, A. P. (2021). Use of dietary phytochemicals for inhibition of trimethylamine N-oxide formation. *The Journal of Nutritional Biochemistry,**91*, Article 108600. 33577949 10.1016/j.jnutbio.2021.108600

[CR25] Jaremek, M., Yu, Z., Mangino, M., et al. (2013). Alcohol-induced metabolomic differences in humans. *Translational Psychiatry,**3*, Article e276. 23820610 10.1038/tp.2013.55 PMC3731787

[CR26] Jiang, W., Hu, J. W., He, X. R., Jin, W. L., & He, X. Y. (2021). Statins: A repurposed drug to fight cancer. *Journal of experimental & clinical cancer research: CR,**40*, 241. 34303383 10.1186/s13046-021-02041-2 PMC8306262

[CR27] Johnson, W. E., Li, C., & Rabinovic, A. (2007). Adjusting batch effects in microarray expression data using empirical Bayes methods. *Biostatistics (Oxford, England),**8*, 118–27. 16632515 10.1093/biostatistics/kxj037

[CR28] Kaddurah-Daouk, R., Baillie, R. A., Zhu, H., et al. (2010). Lipidomic analysis of variation in response to simvastatin in the Cholesterol and Pharmacogenetics Study. *Metabolomics: Official Journal of the Metabolomic Society,**6*, 191–201. 20445760 10.1007/s11306-010-0207-x PMC2862962

[CR29] Kambalapally, C., Suthar, P. K., Patale, P. (2023). Chap. 51 - Trigonelline and its uses in stroke. In: Martin CR, Patel VB, Preedy VR, eds. Treatments, Nutraceuticals, Supplements, and Herbal Medicine in Neurological Disorders: Academic Press. pp. 979 – 92.

[CR30] Khan, S. U., Khan, M. U., Riaz, H. (2019). Effects of Nutritional Supplements and Dietary Interventions on Cardiovascular Outcomes: An Umbrella Review and Evidence Map. Annals of internal medicine. 171: 190-8. 10.7326/M19-0341 PMC7261374 31284304

[CR31] Kolawole, O. R., & Kashfi, K. (2022). NSAIDs and cancer resolution: New paradigms beyond cyclooxygenase. *International journal of molecular sciences*. 10.3390/ijms23031432 35163356 10.3390/ijms23031432 PMC8836048

[CR32] Kuang, A., Erlund, I., Herder, C., Westerhuis, J. A., Tuomilehto, J., & Cornelis, M. C. (2018). Lipidomic response to coffee consumption. *Nutrients*. 10.3390/nu10121851 30513727 10.3390/nu10121851 PMC6315510

[CR33] Langenau, J., Boeing, H., Bergmann, M. M., Nothlings, U., & Oluwagbemigun, K. (2019). The Association between Alcohol Consumption and Serum Metabolites and the Modifying Effect of Smoking. Nutrients. 11. 10.3390/nu11102331 PMC6836136 31581552

[CR34] Lara-Guzmán, O. J., Álvarez, R., & Muñoz-Durango, K. (2021). Changes in the plasma lipidome of healthy subjects after coffee consumption reveal potential cardiovascular benefits: A randomized controlled trial. *Free radical biology & medicine,**176*, 345–55. 34648905 10.1016/j.freeradbiomed.2021.10.012

[CR35] Lee, J., Lee, J-Y., & Kang, H. (2025). Excessive alcohol consumption: a driver of metabolic dysfunction and inflammation. *Frontiers in Toxicology Volume* 7–2025. 10.3389/ftox.2025.1670769 PMC12515849 41090150

[CR36] Li, K., Wang, X. F., Li, D. Y., et al. (2018). The good, the bad, and the ugly of calcium supplementation: A review of calcium intake on human health. *Clinical Interventions in Aging,**13*, 2443–52. 30568435 10.2147/CIA.S157523 PMC6276611

[CR37] Lim, S., Sakuma, I., Quon, M. J., & Koh, K. K. (2014). Differential metabolic actions of specific statins: Clinical and therapeutic considerations. *Antioxidants & Redox Signaling,**20*, 1286–1299. 23924053 10.1089/ars.2013.5531 PMC4692132

[CR38] Liu, D., Meng, X., Tian, Q., et al., et al. (2022). Vitamin D and Multiple Health Outcomes: An Umbrella Review of Observational Studies, Randomized Controlled Trials, and Mendelian Randomization Studies. *Advances in nutrition (Bethesda Md)*, *13*, 1044–1062. 34999745 10.1093/advances/nmab142 PMC9340982

[CR39] Liu, D., Yang, Z., Chandler, K., et al. (2022). Serum metabolomic analysis reveals several novel metabolites in association with excessive alcohol use - an exploratory study. *Translational Research : The Journal Of Laboratory And Clinical Medicine*, *240*, 87–98. 34743014 10.1016/j.trsl.2021.10.008 PMC9506418

[CR40] Matthew, K. A., Getz, K. R., Jeon, M. S., Luo, C., Luo, J., & Toriola, A. T. (2024). Associations of vitamins and related cofactor metabolites with mammographic breast density in premenopausal women. *Journal of Nutrition,**154*, 424–34. 38122846 10.1016/j.tjnut.2023.12.023 PMC10900193

[CR41] Metabolon (2023). Complex Lipids Targeted Panel.

[CR42] Mitro, S. D., Wu, J., Rahman, M. L. (2021). Longitudinal Plasma Metabolomics Profile in Pregnancy-A Study in an Ethnically Diverse U.S. Pregnancy Cohort. *Nutrients* 13. 10.3390/nu13093080 PMC8471130 34578958

[CR43] Poole, R., Kennedy, O. J., Roderick, P., Fallowfield, J. A., Hayes, P. C., & Parkes, J. (2017). Coffee consumption and health: Umbrella review of meta-analyses of multiple health outcomes. *BMJ (Clinical research ed.),**359*, Article j5024. 29167102 10.1136/bmj.j5024 PMC5696634

[CR44] Roerecke, M. (2021). Alcohol’s impact on the cardiovascular system. *Nutrients*. 10.3390/nu13103419 34684419 10.3390/nu13103419 PMC8540436

[CR45] Rumgay, H., Murphy, N., Ferrari, P., & Soerjomataram, I. (2021). Alcohol and cancer: Epidemiology and biological mechanisms. *Nutrients*. 10.3390/nu13093173 34579050 10.3390/nu13093173 PMC8470184

[CR46] Sachse, D., Sletner, L., Mørkrid, K., et al. (2012). Metabolic changes in urine during and after pregnancy in a large, multiethnic population-based cohort study of gestational diabetes. *PLoS One,**7*, Article e52399. 23285025 10.1371/journal.pone.0052399 PMC3528643

[CR47] Sánchez, M. C., Herráiz, A., Ciudad, M. J., et al. (2024). Metabolomics and biochemical benefits of multivitamin and multimineral supplementation in healthy individuals: A pilot study. *Foods,**13*, Article 2207. 39063291 10.3390/foods13142207 PMC11275291

[CR48] Sánchez, M. C., Herráiz, A., Ciudad, M. J., et al. (2024). Metabolomics and biochemical benefits of multivitamin and multimineral supplementation in healthy individuals: A pilot study. *Foods*. 10.3390/foods13142207 39063291 10.3390/foods13142207 PMC11275291

[CR49] Schaibley, V. M., Ramos, I. N., Woosley, R. L., Curry, S., Hays, S., & Ramos, K. S. (2022). Limited genomics training among physicians remains a barrier to genomics-based implementation of precision medicine. *Frontiers in Medicine*. 10.3389/fmed.2022.757212 35372454 10.3389/fmed.2022.757212 PMC8971187

[CR50] Schjerning, A. M., McGettigan, P., & Gislason, G. (2020). Cardiovascular effects and safety of (non-aspirin) NSAIDs. *Nature reviews Cardiology*, *17*, 574–584. 32322101 10.1038/s41569-020-0366-z

[CR51] Stancu, C., & Sima, A. (2001). Statins: Mechanism of action and effects. *Journal of Cellular and Molecular Medicine,**5*, 378–87. 12067471 10.1111/j.1582-4934.2001.tb00172.x PMC6740083

[CR52] Stevens, V. L., Carter, B. D., Jacobs, E. J., McCullough, M. L., Teras, L. R., & Wang, Y. (2023). A prospective case-cohort analysis of plasma metabolites and breast cancer risk. *Breast cancer research: BCR*, *25*, 5. 36650550 10.1186/s13058-023-01602-x PMC9847033

[CR53] Stith, J. L., Velazquez, F. N., & Obeid, L. M. (2019). Advances in determining signaling mechanisms of ceramide and role in disease. *Journal of Lipid Research,**60*, 913–918. 30846529 10.1194/jlr.S092874 PMC6495170

[CR54] Suhre, K., Stephan, N., Zaghlool, S., et al. (2022). Matching drug metabolites from non-targeted metabolomics to self-reported medication in the Qatar Biobank study. *Metabolites*. 10.3390/metabo12030249 35323692 10.3390/metabo12030249 PMC8948833

[CR55] Sullivan, V. K., Chen, J., Bernard, L., et al. (2025). Serum and urine metabolite correlates of vitamin D supplementation in the Atherosclerosis Risk in Communities (ARIC) study. *Clin Nutr ESPEN*, *67*, 523–532. 40189143 10.1016/j.clnesp.2025.03.172 PMC12085285

[CR56] Taylor, F., Huffman, M. D., Macedo, A. F. (2013). Statins for the primary prevention of cardiovascular disease. The Cochrane database of systematic reviews. 2013: Cd004816. 10.1002/14651858.CD004816.pub5 PMC6481400 23440795

[CR57] Toriola, A. T., Appleton, C. M., Zong, X., et al. (2018). Circulating receptor activator of nuclear factor-κB (RANK), RANK ligand (RANKL), and mammographic density in premenopausal women. *Cancer Prevention Research,**11*, 789–96. 30352839 10.1158/1940-6207.CAPR-18-0199 PMC6784533

[CR58] Trompet, S., Postmus, I., Slagboom, P. E., et al. (2016). Non-response to (statin) therapy: The importance of distinguishing non-responders from non-adherers in pharmacogenetic studies. *European Journal of Clinical Pharmacology,**72*, 431–7. 26686871 10.1007/s00228-015-1994-9 PMC4792342

[CR59] van Dam, R. M., Hu, F. B., & Willett, W. C. (2020). Coffee, caffeine, and health. *New England Journal of Medicine*, *383*, 369–378. 32706535 10.1056/NEJMra1816604

[CR60] van der Veen, J. N., Kennelly, J. P., Wan, S., Vance, J. E., Vance, D. E., & Jacobs, R. L. (2017). The critical role of phosphatidylcholine and phosphatidylethanolamine metabolism in health and disease. *Biochimica et Biophysica Acta (BBA),**1859*, 1558–72. 10.1016/j.bbamem.2017.04.006 28411170

[CR61] Vaughan, C. J., & Gotto, A. M., Jr. (2004). Update on statins: 2003. *Circulation,**110*, 886–92. 10.1161/01.CIR.0000139312.10076.BA 15313959

[CR62] Veronese, N., Demurtas, J., Thompson, T., et al. (2020). Effect of low-dose aspirin on health outcomes: An umbrella review of systematic reviews and meta-analyses. *British journal of clinical pharmacology*, *86*, 1465–1475. 32488906 10.1111/bcp.14310 PMC7373714

[CR63] Wajapeyee, N., Beamon, T. C., & Gupta, R. (2024). Roles and therapeutic targeting of ceramide metabolism in cancer. *Molecular Metabolism,**83*, Article 101936. 38599378 10.1016/j.molmet.2024.101936 PMC11031839

[CR64] Wang, L., Zhang, W., Wang, R., et al. (2022). Estimating the time of last drinking from blood ethyl glucuronide and ethyl sulphate concentrations. *Scientific Reports,**12*, Article 14262. 35995832 10.1038/s41598-022-18527-8 PMC9395533

[CR65] Wang, Y., Hodge, R. A., Stevens, V. L., Hartman, T. J., & McCullough, M. L. (2020). Identification and Reproducibility of Plasma Metabolomic Biomarkers of Habitual Food Intake in a US Diet Validation Study. Metabolites. 10. 10.3390/metabo10100382 PMC7600452 32993181

[CR66] Wen, B., Mei, Z., Zeng, C., & Liu, S. (2017). metaX: A flexible and comprehensive software for processing metabolomics data. *BMC Bioinformatics,**18*, 183. 28327092 10.1186/s12859-017-1579-y PMC5361702

[CR67] Wurst, F. M., Skipper, G. E., & Weinmann, W. (2003). Ethyl glucuronide—The direct ethanol metabolite on the threshold from science to routine use. *Addiction,**98*, 51–61. 14984242 10.1046/j.1359-6357.2003.00588.x

[CR68] Yang, Z., Gao, H., Ma, J., et al. (2024). Unique urine and serum metabolomic signature in patients with excessive alcohol use: An exploratory study. *Alcohol, clinical & experimental research,**48*, 1519–28. 10.1111/acer.15398 38951043

[CR69] Yu, Y., Zhang, N., Mai, Y., et al. (2023). Correcting batch effects in large-scale multiomics studies using a reference-material-based ratio method. *Genome biology*, *24*, 201. 37674217 10.1186/s13059-023-03047-z PMC10483871

[CR70] Zakhari, S. (2006). Overview: How is alcohol metabolized by the body? *Alcohol Research & Health: The Journal of the National Institute on Alcohol Abuse and Alcoholism,**29*, 245–254. 17718403 PMC6527027

[CR71] Zeng, C., Rosenberg, L., Li, X., et al. (2022). Sodium-containing acetaminophen and cardiovascular outcomes in individuals with and without hypertension. *European Heart Journal,**43*, 1743–55. 35201347 10.1093/eurheartj/ehac059 PMC9076395

[CR72] Zhu, J., Zhou, L., Zhao, M., Wei, F., Fu, H., & Marchioni, E. (2023). Revealing the dynamic changes of lipids in coffee beans during roasting based on UHPLC-QE-HR-AM/MS/MS. *Food research international (Ottawa, Ont.),**174*, Article 113507. 37986503 10.1016/j.foodres.2023.113507

