# Supplementary material for: Associations of coffee, alcohol, medication and supplement use with the metabolome and lipidome: an observational study of premenopausal women
Source: Metabolomics. 2026 May 24;22(3):85. doi: 10.1007/s11306-026-02467-9 (PMC13199196; doi:10.1007/s11306-026-02467-9)
Supplement: Supplementary file 1 — Supplementary file1 (DOCX 1703 KB) [file 11306_2026_2467_MOESM1_ESM.docx]

**Supplementary Table S1****. Adjusted Least Square Means of Lipid Species and Metabolites Associated with** **Frequency of Alcohol Intake^ab^**

| **Lipid Species** | **Never** | **<1 Drink** | **1-2 Drinks** | **3-5 Drinks** | **≥6 Drinks** | **FDR**  **P_trend_^c^** | **Bonferroni**  **P_trend_^d^** |  |
| --- | --- | --- | --- | --- | --- | --- | --- | --- |
|  | **LSM**  **(95%CI)** | **LSM**  **(95%CI)** | **LSM**  **(95%CI)** | **LSM**  **(95%CI)** | **LSM**  **(95%CI)** |  |  |  |
| **Cholesteryl Esters** |  |  |  |  |  |  |  |  |
| CE(14:0) | 2.91   (2.85, 2.97) | 2.93   (2.87, 3.00) | 2.95   (2.88, 3.03) | 3.03   (2.96, 3.11) | 3.13   (3.01, 3.24) | 3.80E-03 | 2.62E-01 |  |
| CE(14:1) | -0.08   (-0.16, -0.00) | -0.04   (-0.12, 0.05) | 0.00   (-0.10, 0.10) | 0.11   (0.01, 0.21) | 0.29   (0.14, 0.45) | 1.81E-04 | 4.88E-03 |  |
| CE(16:1) | 4.30   (4.24, 4.37) | 4.32   (4.26, 4.39) | 4.39   (4.31, 4.46) | 4.49   (4.41, 4.57) | 4.77   (4.64, 4.89) | 9.72E-10 | 2.92E-09 |  |
| CE(18:3) | 3.86   (3.82, 3.91) | 3.87   (3.82, 3.92) | 3.86   (3.80, 3.92) | 3.92   (3.86, 3.98) | 4.03   (3.93, 4.12) | 7.58E-03 | 6.44E-01 |  |
| CE(18:4) | -0.03   (-0.11, 0.04) | -0.03   (-0.11, 0.04) | 0.00   (-0.09, 0.09) | 0.15   (0.05, 0.24) | 0.36   (0.22, 0.51) | 5.93E-06 | 7.12E-05 |  |
| CE(20:5) | 2.78   (2.70, 2.85) | 2.88   (2.81, 2.95) | 2.92   (2.84, 3.01) | 2.98   (2.89, 3.07) | 3.15   (3.01, 3.29) | 1.34E-04 | 3.34E-03 |  |
| **Ceramides** |  |  |  |  |  |  |  |  |
| CER(24:1) | 0.02   (-0.02, 0.06) | 0.09   (0.05, 0.12) | 0.09   (0.04, 0.13) | 0.12   (0.07, 0.16) | 0.18   (0.11, 0.25) | 3.07E-03 | 1.93E-01 |  |
| CER(26:1) | -3.88   (-3.93, -3.84) | -3.80   (-3.85, -3.76) | -3.76   (-3.82, -3.71) | -3.73   (-3.78, -3.68) | -3.57   (-3.65, -3.49) | 9.29E-08 | 4.65E-07 |  |
| **Diacylglycerols** |  |  |  |  |  |  |  |  |
| DAG(14:0/16:1) | -3.66   (-3.78, -3.54) | -3.60   (-3.73, -3.48) | -3.52   (-3.66, -3.37) | -3.42   (-3.56, -3.27) | -3.23   (-3.46, -3.00) | 5.60E-03 | 4.48E-01 |  |
| DAG(14:0/22:6) | -4.87   (-4.96, -4.78) | -4.78   (-4.87, -4.69) | -4.76   (-4.87, -4.66) | -4.60   (-4.71, -4.49) | -4.49   (-4.66, -4.32) | 4.37E-04 | 1.66E-02 |  |
| DAG(16:0/16:1) | -1.69   (-1.80, -1.57) | -1.65   (-1.77, -1.53) | -1.55   (-1.69, -1.41) | -1.45   (-1.59, -1.31) | -1.24   (-1.46, -1.01) | 2.22E-03 | 1.26E-01 |  |
| DAG(16:0/20:3) | -2.63   (-2.70, -2.55) | -2.58   (-2.66, -2.50) | -2.52   (-2.62, -2.43) | -2.48   (-2.58, -2.39) | -2.33   (-2.48, -2.19) | 5.24E-03 | 4.03E-01 |  |
| DAG(16:0/20:5) | -3.69   (-3.79, -3.60) | -3.59   (-3.69, -3.49) | -3.54   (-3.66, -3.42) | -3.42   (-3.55, -3.30) | -3.20   (-3.39, -3.01) | 1.25E-04 | 3.00E-03 |  |
| DAG(16:0/22:5) | -3.17   (-3.25, -3.08) | -3.04   (-3.13, -2.96) | -3.09   (-3.19, -2.99) | -2.99   (-3.10, -2.89) | -2.84   (-3.00, -2.67) | 5.67E-03 | 4.59E-01 |  |
| DAG(16:1/16:1) | -3.56   (-3.68, -3.44) | -3.53   (-3.66, -3.41) | -3.47   (-3.62, -3.33) | -3.32   (-3.47, -3.17) | -2.98   (-3.22, -2.74) | 1.24E-04 | 2.86E-03 |  |
| DAG(16:1/18:0) | -2.75   (-2.84, -2.66) | -2.74   (-2.83, -2.64) | -2.65   (-2.76, -2.53) | -2.58   (-2.70, -2.47) | -2.37   (-2.55, -2.20) | 1.41E-03 | 7.48E-02 |  |
| DAG(16:1/18:1) | -0.42   (-0.51, -0.32) | -0.36   (-0.46, -0.26) | -0.32   (-0.43, -0.20) | -0.26   (-0.38, -0.14) | -0.05   (-0.24, 0.13) | 5.27E-03 | 4.11E-01 |  |
| DAG(16:1/20:0) | -4.12   (-4.19, -4.05) | -4.05   (-4.12, -3.98) | -3.96   (-4.05, -3.88) | -3.85   (-3.94, -3.77) | -3.74   (-3.87, -3.60) | 7.18E-06 | 1.00E-04 |  |
| DAG(16:1/20:4) | -2.93   (-3.02, -2.83) | -2.92   (-3.02, -2.82) | -2.87   (-2.99, -2.75) | -2.74   (-2.86, -2.62) | -2.53   (-2.72, -2.35) | 6.56E-04 | 2.90E-02 |  |
| DAG(16:1/22:6) | -4.36   (-4.46, -4.25) | -4.27   (-4.38, -4.16) | -4.21   (-4.33, -4.08) | -4.06   (-4.19, -3.93) | -3.88   (-4.08, -3.68) | 2.85E-04 | 9.57E-03 |  |
| DAG(18:1/20:5) | -2.39   (-2.48, -2.30) | -2.29   (-2.38, -2.20) | -2.26   (-2.37, -2.16) | -2.15   (-2.25, -2.04) | -2.06   (-2.22, -1.89) | 3.11E-03 | 1.99E-01 |  |
| **Dihydroceramides** |  |  |  |  |  |  |  |  |
| DCER(22:2) | -1.71   (-1.77, -1.65) | -1.63   (-1.70, -1.57) | -1.57   (-1.65, -1.50) | -1.50   (-1.57, -1.42) | -1.43   (-1.55, -1.31) | 5.60E-04 | 2.24E-02 |  |
| DCER(24:1) | -1.12   (-1.15, -1.08) | -1.04   (-1.08, -1.00) | -1.01   (-1.06, -0.97) | -1.00   (-1.05, -0.96) | -0.91   (-0.98, -0.84) | 1.42E-04 | 3.70E-03 |  |
| **Hexosylceramides** |  |  |  |  |  |  |  |  |
| HCER(26:0) | -4.40   (-4.44, -4.37) | -4.39   (-4.43, -4.35) | -4.39   (-4.43, -4.35) | -4.37   (-4.41, -4.32) | -4.22   (-4.29, -4.16) | 1.13E-04 | 2.48E-03 |  |
| **Lysophosphatidylcholines** |  |  |  |  |  |  |  |  |
| LPC(16:0) | 4.37   (4.34, 4.40) | 4.40   (4.37, 4.43) | 4.42   (4.38, 4.46) | 4.45   (4.41, 4.49) | 4.48   (4.42, 4.54) | 9.06E-03 | 7.97E-01 |  |
| LPC(16:1) | 0.48   (0.43, 0.53) | 0.50   (0.45, 0.55) | 0.52   (0.47, 0.58) | 0.57   (0.51, 0.63) | 0.74   (0.65, 0.83) | 7.20E-06 | 1.08E-04 |  |
| LPC(22:6) | -0.82   (-0.87, -0.77) | -0.82   (-0.87, -0.77) | -0.79   (-0.85, -0.73) | -0.76   (-0.82, -0.70) | -0.65   (-0.75, -0.56) | 7.09E-03 | 5.95E-01 |  |
| **Lysophosphatidylethanolamine** |  |  |  |  |  |  |  |  |
| LPE(22:5) | -1.92   (-1.97, -1.87) | -1.91   (-1.96, -1.86) | -1.90   (-1.96, -1.84) | -1.89   (-1.95, -1.83) | -1.72   (-1.81, -1.62) | 2.50E-03 | 1.52E-01 |  |
| **Phosphatidylcholines** |  |  |  |  |  |  |  |  |
| PC(14:0/18:1) | 1.53   (1.47, 1.59) | 1.57   (1.51, 1.64) | 1.58   (1.51, 1.65) | 1.67   (1.60, 1.74) | 1.77   (1.65, 1.88) | 1.38E-03 | 7.20E-02 |  |
| PC(15:0/18:2) | 0.38   (0.33, 0.42) | 0.45   (0.41, 0.50) | 0.38   (0.33, 0.44) | 0.36   (0.30, 0.41) | 0.20   (0.11, 0.29) | 6.42E-04 | 2.76E-02 |  |
| PC(16:0/14:0) | 0.66   (0.59, 0.73) | 0.69   (0.61, 0.77) | 0.74   (0.65, 0.83) | 0.88   (0.79, 0.97) | 1.06   (0.92, 1.20) | 3.67E-06 | 3.67E-05 |  |
| PC(16:0/16:0) | 2.85   (2.82, 2.88) | 2.88   (2.85, 2.91) | 2.90   (2.87, 2.94) | 2.92   (2.88, 2.96) | 3.01   (2.95, 3.07) | 2.85E-04 | 9.70E-03 |  |
| PC(16:0/16:1) | 2.27   (2.20, 2.35) | 2.30   (2.22, 2.38) | 2.39   (2.30, 2.49) | 2.54   (2.44, 2.64) | 2.92   (2.77, 3.07) | 3.35E-12 | 3.35E-12 |  |
| PC(16:0/18:0) | 2.48   (2.45, 2.51) | 2.51   (2.48, 2.54) | 2.52   (2.49, 2.56) | 2.53   (2.50, 2.57) | 2.63   (2.57, 2.69) | 5.70E-04 | 2.34E-02 |  |
| PC(16:0/18:1) | 5.51   (5.47, 5.55) | 5.56   (5.52, 5.60) | 5.59   (5.54, 5.63) | 5.63   (5.58, 5.68) | 5.77   (5.70, 5.85) | 1.82E-07 | 1.27E-06 |  |
| PC(16:0/18:3) | 2.20   (2.14, 2.25) | 2.20   (2.14, 2.26) | 2.20   (2.13, 2.27) | 2.36   (2.29, 2.44) | 2.49   (2.37, 2.60) | 7.18E-06 | 9.65E-05 |  |
| PC(16:0/20:4) | 5.46   (5.42, 5.50) | 5.52   (5.48, 5.57) | 5.54   (5.49, 5.59) | 5.57   (5.52, 5.62) | 5.69   (5.61, 5.77) | 8.30E-05 | 1.74E-03 |  |
| PC(16:0/20:5) | 2.58   (2.51, 2.66) | 2.71   (2.63, 2.79) | 2.81   (2.72, 2.90) | 2.93   (2.84, 3.02) | 3.14   (2.99, 3.29) | 4.43E-09 | 1.77E-08 |  |
| PC(16:0/22:4) | 2.06   (2.01, 2.11) | 2.06   (2.00, 2.11) | 2.07   (2.01, 2.13) | 2.08   (2.02, 2.15) | 2.27   (2.17, 2.37) | 1.56E-03 | 8.43E-02 |  |
| PC(16:0/22:5) | 3.28   (3.24, 3.33) | 3.32   (3.28, 3.37) | 3.32   (3.27, 3.37) | 3.34   (3.28, 3.39) | 3.49   (3.41, 3.58) | 6.56E-04 | 2.95E-02 |  |
| PC(17:0/18:2) | 1.24   (1.20, 1.28) | 1.34   (1.30, 1.39) | 1.29   (1.24, 1.34) | 1.24   (1.19, 1.29) | 1.10   (1.02, 1.17) | 2.58E-04 | 7.74E-03 |  |
| PC(18:0/16:1) | 0.16   (0.10, 0.23) | 0.19   (0.12, 0.25) | 0.24   (0.17, 0.32) | 0.30   (0.22, 0.38) | 0.66   (0.53, 0.78) | 8.29E-10 | 1.66E-09 |  |
| PC(18:0/18:3) | 0.93   (0.87, 1.00) | 0.92   (0.85, 0.99) | 0.92   (0.84, 1.00) | 1.03   (0.95, 1.11) | 1.21   (1.08, 1.33) | 2.62E-04 | 8.12E-03 |  |
| PC(18:0/20:5) | 1.86   (1.79, 1.94) | 1.96   (1.88, 2.04) | 2.03   (1.94, 2.12) | 2.09   (1.99, 2.18) | 2.31   (2.17, 2.46) | 7.66E-06 | 1.23E-04 |  |
| PC(18:1/16:1) | 2.09   (2.04, 2.14) | 2.13   (2.08, 2.18) | 2.13   (2.07, 2.19) | 2.16   (2.10, 2.22) | 2.31   (2.22, 2.41) | 1.07E-03 | 5.27E-02 |  |
| PC(18:1/20:5) | 0.63   (0.57, 0.69) | 0.74   (0.68, 0.81) | 0.77   (0.69, 0.84) | 0.86   (0.78, 0.94) | 0.94   (0.82, 1.05) | 1.81E-04 | 5.07E-03 |  |
| PC(20:0/18:1) | -0.07   (-0.11, -0.03) | -0.03   (-0.07, 0.01) | -0.02   (-0.06, 0.03) | -0.01   (-0.06, 0.04) | 0.11   (0.04, 0.18) | 6.84E-04 | 3.15E-02 |  |
| **Phosphatidylethanolamines** |  |  |  |  |  |  |  |  |
| PE(18:0/18:3) | -0.63   (-0.68, -0.58) | -0.61   (-0.66, -0.56) | -0.62   (-0.68, -0.56) | -0.56   (-0.62, -0.50) | -0.45   (-0.55, -0.36) | 5.11E-03 | 3.84E-01 |  |
| PE(18:0/20:5) | -0.72   (-0.79, -0.66) | -0.60   (-0.67, -0.53) | -0.57   (-0.65, -0.49) | -0.49   (-0.57, -0.41) | -0.36   (-0.49, -0.24) | 4.16E-05 | 7.49E-04 |  |
| **Phosphatidylinositol** |  |  |  |  |  |  |  |  |
| PI(16:0/20:4) | -1.13   (-1.18, -1.09) | -1.11   (-1.16, -1.07) | -1.11   (-1.16, -1.06) | -1.14   (-1.20, -1.09) | -0.94   (-1.02, -0.85) | 5.60E-03 | 4.43E-01 |  |
| **Sphingomyelins** |  |  |  |  |  |  |  |  |
| SM(26:1) | -0.22   (-0.25, -0.19) | -0.18   (-0.21, -0.14) | -0.18   (-0.22, -0.14) | -0.18   (-0.22, -0.14) | -0.07   (-0.13, -0.00) | 1.65E-03 | 9.06E-02 |  |
| **Triacylglycerols** |  |  |  |  |  |  |  |  |
| TAG46:0-FA14:0 | 0.18   (-0.01, 0.36) | 0.19   (-0.00, 0.38) | 0.41   (0.19, 0.64) | 0.58   (0.35, 0.80) | 0.81   (0.46, 1.16) | 4.67E-03 | 3.45E-01 |  |
| TAG46:0-FA16:0 | 0.90   (0.71, 1.08) | 0.92   (0.73, 1.11) | 1.12   (0.90, 1.35) | 1.32   (1.10, 1.55) | 1.55   (1.20, 1.90) | 3.67E-03 | 2.39E-01 |  |
| TAG46:1-FA14:1 | -1.65   (-1.82, -1.48) | -1.66   (-1.84, -1.48) | -1.45   (-1.66, -1.24) | -1.27   (-1.48, -1.06) | -1.07   (-1.40, -0.73) | 4.67E-03 | 3.41E-01 |  |
| TAG46:1-FA16:1 | -0.32   (-0.49, -0.15) | -0.32   (-0.50, -0.14) | -0.15   (-0.36, 0.06) | 0.02   (-0.20, 0.24) | 0.29   (-0.05, 0.62) | 4.39E-03 | 3.11E-01 |  |
| TAG48:0-FA16:0 | 2.29   (2.14, 2.44) | 2.28   (2.12, 2.43) | 2.50   (2.31, 2.68) | 2.65   (2.47, 2.84) | 2.91   (2.62, 3.20) | 5.85E-04 | 2.46E-02 |  |
| TAG48:1-FA16:0 | 2.76   (2.63, 2.90) | 2.79   (2.65, 2.93) | 2.89   (2.73, 3.06) | 3.03   (2.86, 3.20) | 3.22   (2.96, 3.48) | 7.98E-03 | 6.86E-01 |  |
| TAG48:1-FA16:1 | 1.15   (1.01, 1.30) | 1.14   (0.99, 1.29) | 1.33   (1.15, 1.50) | 1.47   (1.29, 1.65) | 1.78   (1.50, 2.07) | 3.69E-04 | 1.33E-02 |  |
| TAG48:2-FA16:1 | 1.49   (1.36, 1.63) | 1.51   (1.37, 1.65) | 1.61   (1.45, 1.78) | 1.72   (1.55, 1.89) | 2.02   (1.76, 2.28) | 2.45E-03 | 1.47E-01 |  |
| TAG50:0-FA16:0 | 2.32   (2.20, 2.44) | 2.33   (2.21, 2.46) | 2.48   (2.34, 2.63) | 2.57   (2.42, 2.72) | 2.75   (2.52, 2.98) | 4.46E-03 | 3.21E-01 |  |
| TAG50:0-FA18:0 | 1.29   (1.16, 1.42) | 1.30   (1.16, 1.43) | 1.47   (1.32, 1.63) | 1.59   (1.42, 1.75) | 1.77   (1.52, 2.03) | 2.91E-03 | 1.80E-01 |  |
| TAG50:1-FA16:1 | 1.09   (0.97, 1.20) | 1.11   (0.99, 1.23) | 1.24   (1.10, 1.38) | 1.32   (1.18, 1.46) | 1.60   (1.37, 1.82) | 5.28E-04 | 2.06E-02 |  |
| TAG50:2-FA16:1 | 3.18   (3.07, 3.28) | 3.21   (3.11, 3.32) | 3.30   (3.18, 3.43) | 3.35   (3.22, 3.48) | 3.59   (3.38, 3.79) | 3.78E-03 | 2.57E-01 |  |
| TAG50:4-FA14:0 | 1.82   (1.73, 1.92) | 1.88   (1.77, 1.98) | 1.81   (1.69, 1.93) | 1.76   (1.64, 1.88) | 1.47   (1.28, 1.66) | 4.22E-03 | 2.95E-01 |  |
| TAG51:2-FA15:0 | 0.90   (0.82, 0.98) | 1.01   (0.93, 1.10) | 0.98   (0.88, 1.07) | 0.85   (0.75, 0.95) | 0.68   (0.52, 0.83) | 6.48E-03 | 5.32E-01 |  |
| TAG51:3-FA15:0 | 1.03   (0.96, 1.11) | 1.15   (1.07, 1.23) | 1.06   (0.96, 1.15) | 0.93   (0.84, 1.03) | 0.63   (0.48, 0.78) | 1.09E-06 | 9.82E-06 |  |
| TAG51:3-FA18:1 | 1.10   (1.02, 1.18) | 1.23   (1.14, 1.31) | 1.15   (1.05, 1.24) | 1.03   (0.93, 1.13) | 0.83   (0.68, 0.99) | 9.24E-04 | 4.34E-02 |  |
| TAG51:3-FA18:2 | 1.47   (1.39, 1.55) | 1.58   (1.50, 1.66) | 1.51   (1.42, 1.60) | 1.43   (1.33, 1.53) | 1.23   (1.08, 1.38) | 3.68E-03 | 2.44E-01 |  |
| TAG51:4-FA15:0 | 0.18   (0.10, 0.26) | 0.30   (0.22, 0.39) | 0.19   (0.09, 0.29) | 0.05   (-0.05, 0.15) | -0.29   (-0.45, -0.13) | 1.36E-07 | 8.14E-07 |  |
| TAG51:4-FA18:1 | -0.95   (-1.03, -0.87) | -0.85   (-0.94, -0.77) | -0.91   (-1.01, -0.82) | -1.01   (-1.11, -0.91) | -1.23   (-1.38, -1.08) | 1.19E-03 | 5.97E-02 |  |
| TAG51:4-FA18:2 | 0.71   (0.63, 0.79) | 0.83   (0.74, 0.91) | 0.72   (0.62, 0.81) | 0.57   (0.47, 0.68) | 0.27   (0.11, 0.42) | 3.44E-07 | 2.75E-06 |  |
| TAG51:5-FA18:2 | -1.42   (-1.50, -1.34) | -1.34   (-1.42, -1.25) | -1.44   (-1.54, -1.34) | -1.52   (-1.63, -1.42) | -1.81   (-1.98, -1.65) | 5.60E-05 | 1.12E-03 |  |
| TAG51:5-FA18:3 | -1.54   (-1.63, -1.46) | -1.44   (-1.53, -1.35) | -1.55   (-1.66, -1.45) | -1.62   (-1.72, -1.51) | -1.86   (-2.03, -1.69) | 1.30E-03 | 6.63E-02 |  |
| TAG52:1-FA16:1 | -1.23   (-1.33, -1.12) | -1.21   (-1.32, -1.10) | -1.09   (-1.21, -0.96) | -1.05   (-1.18, -0.92) | -0.81   (-1.01, -0.61) | 2.32E-03 | 1.35E-01 |  |
| TAG52:5-FA20:5 | -1.19   (-1.31, -1.07) | -1.13   (-1.25, -1.00) | -1.01   (-1.15, -0.86) | -0.84   (-0.98, -0.69) | -0.66   (-0.89, -0.43) | 2.58E-04 | 7.51E-03 |  |
| TAG52:6-FA20:5 | -1.12   (-1.24, -1.01) | -1.07   (-1.19, -0.95) | -0.98   (-1.12, -0.83) | -0.80   (-0.95, -0.66) | -0.61   (-0.84, -0.39) | 2.85E-04 | 9.45E-03 |  |
| TAG53:3-FA17:0 | 1.22   (1.15, 1.29) | 1.33   (1.26, 1.41) | 1.28   (1.19, 1.36) | 1.16   (1.07, 1.25) | 0.92   (0.78, 1.05) | 5.46E-05 | 1.04E-03 |  |
| TAG53:3-FA18:2 | 1.26   (1.18, 1.33) | 1.37   (1.29, 1.44) | 1.31   (1.23, 1.40) | 1.21   (1.12, 1.30) | 0.98   (0.84, 1.12) | 3.96E-04 | 1.46E-02 |  |
| TAG53:4-FA17:0 | 0.37   (0.30, 0.45) | 0.48   (0.40, 0.56) | 0.39   (0.30, 0.49) | 0.27   (0.18, 0.37) | -0.01   (-0.16, 0.14) | 4.59E-06 | 5.05E-05 |  |
| TAG53:4-FA18:2 | 1.18   (1.11, 1.26) | 1.29   (1.21, 1.37) | 1.20   (1.11, 1.29) | 1.09   (1.00, 1.19) | 0.85   (0.70, 0.99) | 4.05E-05 | 6.88E-04 |  |
| TAG54:5-FA18:2 | 3.92   (3.83, 4.01) | 4.00   (3.90, 4.09) | 3.88   (3.77, 3.99) | 3.82   (3.71, 3.93) | 3.62   (3.45, 3.79) | 3.68E-03 | 2.47E-01 |  |
| TAG54:5-FA20:5 | -1.51   (-1.61, -1.42) | -1.43   (-1.53, -1.33) | -1.34   (-1.46, -1.22) | -1.22   (-1.34, -1.10) | -1.07   (-1.25, -0.88) | 2.97E-04 | 1.04E-02 |  |
| TAG54:6-FA20:5 | 0.50   (0.41, 0.59) | 0.58   (0.49, 0.68) | 0.65   (0.53, 0.76) | 0.75   (0.64, 0.87) | 0.87   (0.69, 1.05) | 2.45E-03 | 1.47E-01 |  |
| TAG54:6-FA22:5 | -0.32   (-0.41, -0.22) | -0.27   (-0.37, -0.17) | -0.23   (-0.35, -0.11) | -0.11   (-0.23, 0.01) | 0.00   (-0.18, 0.19) | 8.84E-03 | 7.69E-01 |  |
| TAG54:6-FA22:6 | -0.34   (-0.47, -0.21) | -0.26   (-0.39, -0.12) | -0.13   (-0.28, 0.03) | 0.04   (-0.12, 0.19) | 0.10   (-0.14, 0.34) | 5.17E-03 | 3.93E-01 |  |
| TAG54:7-FA22:6 | -0.25   (-0.37, -0.13) | -0.17   (-0.29, -0.04) | -0.07   (-0.22, 0.07) | 0.08   (-0.07, 0.23) | 0.17   (-0.07, 0.40) | 6.69E-03 | 5.56E-01 |  |
| TAG56:6-FA20:5 | -1.04   (-1.12, -0.95) | -0.91   (-1.00, -0.83) | -0.85   (-0.95, -0.75) | -0.77   (-0.87, -0.67) | -0.68   (-0.84, -0.52) | 1.74E-03 | 9.75E-02 |  |
| TAG56:6-FA22:6 | -0.64   (-0.74, -0.53) | -0.52   (-0.63, -0.41) | -0.42   (-0.55, -0.29) | -0.31   (-0.44, -0.18) | -0.26   (-0.47, -0.05) | 9.75E-03 | 8.67E-01 |  |
| TAG58:10-FA20:5 | -1.89   (-1.99, -1.79) | -1.82   (-1.92, -1.71) | -1.72   (-1.84, -1.60) | -1.60   (-1.72, -1.48) | -1.48   (-1.67, -1.29) | 1.02E-03 | 4.88E-02 |  |
| **Metabolite** |  |  |  |  |  |  |  |  |
| **Androgenic Steroids** |  |  |  |  |  |  |  |  |
| 5alpha-Androstan-3alpha,17beta-Diol Disulfate | -0.03   (-0.12, 0.07) | -0.04   (-0.14, 0.06) | 0.13   (0.01, 0.24) | 0.21   (0.10, 0.33) | 0.34   (0.15, 0.52) | 1.23E-03 | 7.42E-02 |  |
| 5alpha-Androstan-3alpha,17beta-Diol Monosulfate (2) | -0.15   (-0.24, -0.06) | -0.06   (-0.15, 0.03) | 0.09   (-0.02, 0.20) | 0.30   (0.19, 0.42) | 0.52   (0.35, 0.70) | 2.41E-11 | 2.41E-10 |  |
| 5alpha-Androstan-3beta,17beta-Diol Disulfate | -0.35   (-0.48, -0.21) | -0.21   (-0.35, -0.08) | 0.00   (-0.16, 0.16) | 0.44   (0.27, 0.60) | 0.75   (0.49, 1.00) | 3.46E-14 | 1.73E-13 |  |
| 5alpha-Androstan-3beta,17beta-Diol Monosulfate (2) | -0.12   (-0.23, -0.02) | -0.11   (-0.22, 0.01) | 0.01   (-0.13, 0.14) | 0.20   (0.07, 0.33) | 0.20   (-0.01, 0.41) | 5.37E-03 | 5.80E-01 |  |
| Androstenediol (3beta,17beta) Disulfate (1) | -0.29   (-0.41, -0.17) | -0.13   (-0.26, -0.01) | -0.07   (-0.21, 0.08) | 0.39   (0.24, 0.54) | 0.77   (0.54, 1.01) | 1.04E-15 | 3.13E-15 |  |
| Androstenediol (3beta,17beta) Disulfate (2) | -0.16   (-0.25, -0.08) | -0.04   (-0.13, 0.05) | -0.04   (-0.14, 0.07) | 0.02   (-0.09, 0.12) | 0.17   (0.01, 0.33) | 6.46E-03 | 7.30E-01 |  |
| Androstenediol (3beta,17beta) Monosulfate (1) | -0.24   (-0.34, -0.13) | -0.06   (-0.17, 0.05) | -0.13   (-0.26, -0.00) | 0.08   (-0.05, 0.21) | 0.19   (-0.02, 0.39) | 1.86E-03 | 1.42E-01 |  |
| Androstenediol (3beta,17beta) Monosulfate (2) | -0.19   (-0.29, -0.09) | -0.03   (-0.13, 0.08) | 0.01   (-0.11, 0.13) | 0.31   (0.19, 0.43) | 0.66   (0.46, 0.85) | 5.56E-14 | 3.89E-13 |  |
| Etiocholanolone Glucuronide | -0.15   (-0.24, -0.06) | -0.02   (-0.12, 0.07) | -0.02   (-0.13, 0.09) | 0.19   (0.07, 0.30) | 0.15   (-0.03, 0.32) | 5.51E-03 | 6.06E-01 |  |
| **Benzoate** |  |  |  |  |  |  |  |  |
| 3-Methyl Catechol Sulfate (1) | -0.32   (-0.52, -0.11) | -0.20   (-0.42, 0.01) | 0.22   (-0.03, 0.47) | 0.29   (0.04, 0.55) | 0.42   (0.02, 0.81) | 5.16E-03 | 5.42E-01 |  |
| O-Cresol Sulfate | -0.07   (-0.24, 0.10) | 0.06   (-0.11, 0.23) | 0.29   (0.09, 0.49) | 0.40   (0.20, 0.61) | 0.73   (0.41, 1.06) | 2.02E-04 | 7.69E-03 |  |
| **Chemical** |  |  |  |  |  |  |  |  |
| 1,2,3-Benzenetriol Sulfate (2) | -0.25   (-0.43, -0.07) | -0.14   (-0.33, 0.04) | 0.05   (-0.17, 0.27) | 0.13   (-0.09, 0.35) | 0.51   (0.16, 0.85) | 1.25E-03 | 7.81E-02 |  |
| 3,5-Dichloro-2,6-Dihydroxybenzoic Acid | 0.05   (-0.00, 0.11) | 0.07   (0.01, 0.13) | 0.02   (-0.05, 0.09) | -0.09   (-0.16, -0.01) | -0.17   (-0.28, -0.06) | 3.98E-04 | 1.79E-02 |  |
| Ethyl Glucuronide | -0.18   (-0.29, -0.07) | -0.11   (-0.22, 0.00) | 0.18   (0.05, 0.32) | 0.39   (0.25, 0.52) | 0.99   (0.78, 1.20) | 1.21E-20 | 1.21E-20 |  |
| Ethylparaben Sulfate | 0.20   (-0.05, 0.45) | 0.25   (-0.01, 0.51) | 0.44   (0.13, 0.74) | 0.87   (0.55, 1.18) | 1.04   (0.55, 1.52) | 2.92E-03 | 2.78E-01 |  |
| **Dipeptide** |  |  |  |  |  |  |  |  |
| Cyclo(Leu-Pro) | -0.18   (-0.27, -0.08) | -0.19   (-0.29, -0.09) | 0.08   (-0.03, 0.20) | 0.11   (-0.00, 0.23) | 0.16   (-0.02, 0.34) | 2.49E-03 | 2.19E-01 |  |
| **Endocannabinoid** |  |  |  |  |  |  |  |  |
| N-Oleoyltaurine | -0.17   (-0.25, -0.10) | 0.06   (-0.02, 0.14) | 0.06   (-0.04, 0.15) | 0.01   (-0.09, 0.11) | 0.24   (0.09, 0.39) | 2.21E-03 | 1.78E-01 |  |
| N-Palmitoyltaurine | -0.14   (-0.20, -0.08) | 0.01   (-0.05, 0.08) | 0.02   (-0.06, 0.09) | -0.02   (-0.10, 0.05) | 0.19   (0.08, 0.31) | 1.23E-03 | 7.51E-02 |  |
| Palmitoyl Ethanolamide | -0.08   (-0.12, -0.04) | 0.00   (-0.04, 0.04) | -0.02   (-0.07, 0.03) | 0.04   (-0.01, 0.09) | 0.10   (0.03, 0.18) | 8.75E-04 | 4.56E-02 |  |
| **Fatty Acid** |  |  |  |  |  |  |  |  |
| (16 or 17 or (A19:0 or I19:0) | -0.22   (-0.30, -0.13) | 0.05   (-0.03, 0.14) | 0.07   (-0.03, 0.18) | 0.02   (-0.08, 0.13) | 0.25   (0.09, 0.41) | 1.42E-03 | 9.66E-02 |  |
| (R)-3-Hydroxybutyrylcarnitine | -0.09   (-0.20, 0.01) | 0.07   (-0.03, 0.18) | 0.09   (-0.03, 0.22) | 0.21   (0.08, 0.34) | 0.38   (0.17, 0.58) | 1.07E-03 | 6.09E-02 |  |
| 10-Undecenoate (11:1n1) | -0.22   (-0.29, -0.15) | 0.01   (-0.06, 0.09) | -0.01   (-0.09, 0.08) | 0.03   (-0.06, 0.12) | 0.13   (-0.00, 0.27) | 2.21E-03 | 1.85E-01 |  |
| 16-Hydroxypalmitate | -0.09   (-0.16, -0.03) | 0.02   (-0.05, 0.08) | 0.11   (0.03, 0.18) | 0.07   (-0.01, 0.15) | 0.29   (0.17, 0.41) | 6.18E-05 | 1.24E-03 |  |
| 2-Hydroxyglutarate | -0.07   (-0.12, -0.02) | -0.03   (-0.08, 0.02) | -0.03   (-0.09, 0.03) | 0.11   (0.04, 0.17) | 0.18   (0.08, 0.28) | 1.57E-05 | 2.67E-04 |  |
| 2-Hydroxyphytanate* | -0.16   (-0.25, -0.07) | 0.03   (-0.06, 0.13) | 0.04   (-0.07, 0.15) | 0.09   (-0.02, 0.20) | 0.28   (0.11, 0.45) | 1.07E-03 | 6.19E-02 |  |
| 2s,3r-Dihydroxybutyrate | -0.10   (-0.16, -0.03) | -0.01   (-0.07, 0.06) | 0.02   (-0.06, 0.09) | 0.16   (0.08, 0.23) | 0.24   (0.12, 0.36) | 6.48E-06 | 9.07E-05 |  |
| 3-Hydroxydecanoate | -0.10   (-0.19, -0.02) | -0.02   (-0.11, 0.07) | 0.08   (-0.03, 0.18) | 0.07   (-0.04, 0.18) | 0.25   (0.08, 0.42) | 4.87E-03 | 5.01E-01 |  |
| 3-Hydroxydecanoylcarnitine | -0.19   (-0.27, -0.10) | -0.06   (-0.14, 0.03) | 0.01   (-0.09, 0.11) | 0.01   (-0.09, 0.12) | 0.26   (0.09, 0.42) | 3.15E-04 | 1.29E-02 |  |
| 3-Hydroxymyristate | -0.11   (-0.19, -0.03) | 0.01   (-0.07, 0.10) | 0.10   (-0.01, 0.20) | 0.13   (0.02, 0.23) | 0.27   (0.11, 0.43) | 1.35E-03 | 9.06E-02 |  |
| 3-Hydroxyoctanoylcarnitine (1) | -0.19   (-0.27, -0.11) | -0.06   (-0.14, 0.02) | -0.01   (-0.11, 0.09) | 0.02   (-0.08, 0.12) | 0.29   (0.14, 0.45) | 1.28E-05 | 2.04E-04 |  |
| 3-Hydroxyoctanoylcarnitine (2) | -0.19   (-0.27, -0.11) | -0.06   (-0.14, 0.02) | -0.04   (-0.13, 0.06) | -0.01   (-0.11, 0.09) | 0.25   (0.09, 0.40) | 1.34E-04 | 4.14E-03 |  |
| 3-Hydroxyoleate* | -0.29   (-0.40, -0.18) | -0.15   (-0.27, -0.03) | -0.10   (-0.23, 0.04) | 0.05   (-0.09, 0.19) | 0.25   (0.03, 0.47) | 1.74E-04 | 6.10E-03 |  |
| 3-Hydroxyoleoylcarnitine | -0.03   (-0.09, 0.04) | 0.05   (-0.02, 0.12) | -0.01   (-0.08, 0.07) | -0.01   (-0.09, 0.07) | 0.27   (0.14, 0.39) | 3.02E-03 | 2.96E-01 |  |
| 3-Hydroxystearate | -0.15   (-0.20, -0.10) | -0.04   (-0.09, 0.01) | 0.02   (-0.04, 0.08) | 0.08   (0.01, 0.14) | 0.20   (0.11, 0.30) | 3.66E-08 | 4.03E-07 |  |
| 5-Dodecenoate (12:1n7) | -0.18   (-0.29, -0.07) | 0.07   (-0.04, 0.19) | 0.06   (-0.08, 0.19) | 0.03   (-0.11, 0.16) | 0.32   (0.10, 0.53) | 9.13E-03 | 1.00E+00 |  |
| 9-Hydroxystearate | -0.18   (-0.26, -0.09) | 0.03   (-0.06, 0.12) | -0.01   (-0.11, 0.10) | 0.06   (-0.05, 0.17) | 0.32   (0.16, 0.49) | 7.29E-05 | 1.68E-03 |  |
| N-Palmitoylglycine | -0.16   (-0.22, -0.10) | 0.00   (-0.06, 0.07) | -0.02   (-0.09, 0.05) | -0.04   (-0.11, 0.03) | 0.14   (0.03, 0.25) | 2.87E-03 | 2.64E-01 |  |
| Acetylcarnitine (C2) | -0.03   (-0.07, 0.01) | 0.02   (-0.02, 0.06) | 0.03   (-0.02, 0.08) | 0.04   (-0.01, 0.09) | 0.16   (0.08, 0.23) | 1.48E-03 | 1.02E-01 |  |
| Adrenate (22:4n6) | -0.27   (-0.37, -0.17) | -0.02   (-0.12, 0.08) | -0.11   (-0.22, 0.01) | -0.06   (-0.18, 0.07) | 0.18   (-0.01, 0.36) | 4.87E-03 | 5.02E-01 |  |
| Decadienedioic Acid (C10:2-Dc)** | -0.20   (-0.33, -0.08) | -0.06   (-0.19, 0.07) | -0.05   (-0.20, 0.10) | -0.09   (-0.25, 0.06) | 0.31   (0.07, 0.55) | 8.51E-03 | 1.00E+00 |  |
| Docosadienoate (22:2n6) | -0.18   (-0.25, -0.10) | 0.01   (-0.06, 0.09) | -0.01   (-0.10, 0.09) | 0.02   (-0.08, 0.11) | 0.20   (0.05, 0.35) | 1.63E-03 | 1.21E-01 |  |
| Docosahexaenoate (Dha; 22:6n3) | -0.17   (-0.26, -0.08) | 0.02   (-0.07, 0.11) | 0.04   (-0.06, 0.15) | 0.07   (-0.03, 0.18) | 0.23   (0.07, 0.40) | 2.21E-03 | 1.79E-01 |  |
| Docosapentaenoate (N3 Dpa; 22:5n3) | -0.25   (-0.35, -0.15) | 0.02   (-0.08, 0.12) | -0.00   (-0.12, 0.11) | 0.04   (-0.08, 0.16) | 0.32   (0.14, 0.51) | 1.01E-04 | 2.64E-03 |  |
| Docosapentaenoate (N6 Dpa; 22:5n6) | -0.15   (-0.22, -0.07) | 0.00   (-0.08, 0.08) | -0.03   (-0.12, 0.07) | 0.02   (-0.07, 0.12) | 0.20   (0.05, 0.35) | 2.66E-03 | 2.40E-01 |  |
| Eicosapentaenoate (Epa; 20:5n3) | -0.22   (-0.31, -0.12) | 0.04   (-0.06, 0.14) | 0.05   (-0.06, 0.17) | 0.13   (0.02, 0.25) | 0.45   (0.27, 0.63) | 4.30E-07 | 5.59E-06 |  |
| Erucate (22:1n9) | -0.16   (-0.24, -0.08) | 0.03   (-0.05, 0.11) | -0.02   (-0.12, 0.08) | 0.02   (-0.08, 0.12) | 0.19   (0.03, 0.34) | 6.89E-03 | 8.13E-01 |  |
| Hexadecanedioate (C16-Dc) | -0.06   (-0.14, 0.02) | 0.05   (-0.03, 0.14) | 0.15   (0.05, 0.25) | 0.01   (-0.09, 0.11) | 0.32   (0.17, 0.48) | 6.82E-03 | 7.98E-01 |  |
| Maleate | 0.06   (-0.03, 0.16) | 0.09   (-0.01, 0.19) | 0.21   (0.09, 0.32) | 0.31   (0.19, 0.43) | 0.45   (0.27, 0.64) | 7.87E-04 | 3.96E-02 |  |
| Mead Acid (20:3n9) | -0.15   (-0.22, -0.08) | -0.02   (-0.10, 0.05) | -0.00   (-0.09, 0.09) | 0.00   (-0.09, 0.09) | 0.28   (0.14, 0.42) | 7.29E-05 | 1.62E-03 |  |
| Myristoleate (14:1n5) | -0.30   (-0.42, -0.17) | 0.02   (-0.11, 0.15) | -0.03   (-0.18, 0.12) | -0.01   (-0.17, 0.14) | 0.32   (0.08, 0.56) | 2.21E-03 | 1.81E-01 |  |
| Myristoylcarnitine (C14) | -0.11   (-0.17, -0.05) | 0.03   (-0.03, 0.10) | 0.02   (-0.06, 0.09) | -0.03   (-0.11, 0.04) | 0.19   (0.07, 0.31) | 5.28E-03 | 5.65E-01 |  |
| Palmitate (16:0) | -0.17   (-0.23, -0.10) | 0.01   (-0.05, 0.08) | -0.00   (-0.08, 0.07) | -0.02   (-0.09, 0.06) | 0.12   (0.00, 0.24) | 8.39E-03 | 9.98E-01 |  |
| Palmitoleate (16:1n7) | -0.40   (-0.54, -0.27) | -0.03   (-0.17, 0.11) | -0.12   (-0.28, 0.05) | -0.07   (-0.23, 0.10) | 0.35   (0.09, 0.61) | 3.32E-04 | 1.46E-02 |  |
| Palmitoleoylcarnitine (C16:1)* | -0.12   (-0.18, -0.05) | 0.02   (-0.05, 0.09) | -0.02   (-0.10, 0.06) | -0.06   (-0.14, 0.03) | 0.23   (0.10, 0.36) | 2.21E-03 | 1.85E-01 |  |
| Palmitoylcarnitine (C16) | -0.07   (-0.11, -0.03) | 0.01   (-0.04, 0.05) | -0.00   (-0.05, 0.05) | -0.00   (-0.05, 0.05) | 0.17   (0.09, 0.25) | 1.87E-04 | 6.72E-03 |  |
| Stearidonate (18:4n3) | -0.21   (-0.33, -0.10) | 0.02   (-0.10, 0.14) | -0.05   (-0.19, 0.09) | 0.02   (-0.12, 0.16) | 0.33   (0.11, 0.55) | 1.57E-03 | 1.14E-01 |  |
| Tetradecadienedioate (C14:2-Dc)* | -0.11   (-0.20, -0.01) | 0.04   (-0.06, 0.14) | 0.08   (-0.04, 0.19) | 0.01   (-0.11, 0.13) | 0.42   (0.23, 0.60) | 6.36E-04 | 3.12E-02 |  |
| Tetradecanedioate (C14-Dc) | -0.07   (-0.15, 0.02) | 0.08   (-0.01, 0.17) | 0.13   (0.02, 0.24) | 0.03   (-0.08, 0.14) | 0.35   (0.18, 0.52) | 5.28E-03 | 5.62E-01 |  |
| **Food Component/Plant** |  |  |  |  |  |  |  |  |
| 2,3-Dihydroxyisovalerate | -0.11   (-0.28, 0.07) | -0.14   (-0.32, 0.05) | 0.19   (-0.03, 0.40) | 0.97   (0.76, 1.19) | 1.13   (0.79, 1.46) | 1.34E-13 | 1.21E-12 |  |
| 3-Formylindole | -0.06   (-0.10, -0.01) | 0.00   (-0.04, 0.05) | 0.00   (-0.05, 0.06) | 0.09   (0.03, 0.15) | 0.11   (0.03, 0.20) | 2.22E-03 | 1.91E-01 |  |
| Ergothioneine | -0.23   (-0.32, -0.14) | -0.03   (-0.11, 0.06) | 0.01   (-0.10, 0.11) | 0.14   (0.04, 0.25) | 0.28   (0.11, 0.45) | 1.18E-05 | 1.76E-04 |  |
| Ethyl Alpha-Glucopyranoside | -0.06   (-0.20, 0.08) | -0.00   (-0.15, 0.14) | 0.27   (0.10, 0.44) | 0.74   (0.57, 0.91) | 1.28   (1.02, 1.55) | 1.66E-19 | 3.31E-19 |  |
| Ethyl Beta-Glucopyranoside | -0.10   (-0.27, 0.06) | -0.16   (-0.33, 0.00) | -0.01   (-0.21, 0.18) | 0.71   (0.50, 0.91) | 1.15   (0.84, 1.46) | 7.37E-15 | 2.95E-14 |  |
| Gluconate | 0.04   (0.00, 0.08) | 0.01   (-0.03, 0.05) | -0.03   (-0.07, 0.02) | -0.08   (-0.13, -0.03) | -0.11   (-0.18, -0.03) | 1.57E-03 | 1.15E-01 |  |
| Glucuronide of Piperine Metabolite C17h21no3 (3)* | -0.28   (-0.39, -0.17) | -0.12   (-0.23, -0.00) | 0.01   (-0.12, 0.15) | 0.12   (-0.01, 0.26) | 0.27   (0.06, 0.49) | 1.42E-04 | 4.55E-03 |  |
| Glucuronide of Piperine Metabolite C17h21no3 (4)* | -0.26   (-0.37, -0.15) | -0.10   (-0.22, 0.01) | 0.01   (-0.13, 0.14) | 0.10   (-0.03, 0.24) | 0.22   (0.01, 0.44) | 1.07E-03 | 6.12E-02 |  |
| Glucuronide of Piperine Metabolite C17h21no3 (5)* | -0.19   (-0.30, -0.08) | -0.05   (-0.17, 0.06) | -0.02   (-0.15, 0.12) | 0.14   (-0.00, 0.28) | 0.29   (0.08, 0.51) | 8.75E-04 | 4.64E-02 |  |
| Histidine Betaine (Hercynine)* | -0.18   (-0.26, -0.10) | -0.04   (-0.12, 0.05) | -0.05   (-0.15, 0.05) | 0.03   (-0.07, 0.13) | 0.16   (0.01, 0.32) | 2.89E-03 | 2.71E-01 |  |
| Levulinate (4-Oxovalerate) | -0.05   (-0.13, 0.04) | -0.03   (-0.11, 0.06) | 0.04   (-0.06, 0.14) | 0.11   (0.01, 0.22) | 0.29   (0.12, 0.45) | 1.52E-03 | 1.06E-01 |  |
| Methyl Glucopyranoside (Alpha + Beta) | 0.04   (-0.08, 0.16) | 0.03   (-0.10, 0.15) | 0.05   (-0.09, 0.20) | 0.25   (0.11, 0.40) | 0.48   (0.25, 0.71) | 1.07E-03 | 5.94E-02 |  |
| Piperine | -0.32   (-0.48, -0.17) | -0.14   (-0.30, 0.02) | -0.10   (-0.28, 0.09) | 0.19   (0.00, 0.38) | 0.24   (-0.05, 0.54) | 2.22E-03 | 1.91E-01 |  |
| Quinate | -1.12   (-1.40, -0.85) | -0.59   (-0.88, -0.30) | -0.24   (-0.57, 0.10) | 0.09   (-0.25, 0.43) | 0.17   (-0.36, 0.70) | 4.04E-04 | 1.89E-02 |  |
| Sulfate of Piperine Metabolite C16h19no3 (2)* | -0.30   (-0.42, -0.18) | -0.11   (-0.24, 0.01) | -0.10   (-0.25, 0.04) | 0.14   (-0.01, 0.29) | 0.09   (-0.14, 0.32) | 9.38E-03 | 1.00E+00 |  |
| Sulfate of Piperine Metabolite C18h21no3 (1)* | -0.32   (-0.43, -0.20) | -0.16   (-0.28, -0.05) | -0.13   (-0.27, 0.01) | 0.11   (-0.03, 0.25) | 0.09   (-0.13, 0.31) | 2.78E-03 | 2.53E-01 |  |
| **Glutamate** |  |  |  |  |  |  |  |  |
| 4-Hydroxyglutamate | 0.08   (0.00, 0.17) | 0.11   (0.02, 0.19) | 0.00   (-0.10, 0.10) | -0.13   (-0.23, -0.03) | -0.15   (-0.31, 0.01) | 6.74E-03 | 7.75E-01 |  |
| N-Acetylglutamine | -0.04   (-0.09, 0.01) | -0.02   (-0.07, 0.03) | 0.04   (-0.02, 0.10) | 0.05   (-0.01, 0.11) | 0.17   (0.07, 0.26) | 1.65E-03 | 1.24E-01 |  |
| **Glutathione** |  |  |  |  |  |  |  |  |
| 2-Aminobutyrate | -0.07   (-0.12, -0.02) | -0.01   (-0.06, 0.05) | 0.04   (-0.02, 0.10) | 0.10   (0.04, 0.17) | 0.19   (0.09, 0.29) | 6.65E-05 | 1.40E-03 |  |
| 2-Hydroxybutyrate/2-Hydroxyisobutyrate | -0.14   (-0.22, -0.06) | 0.03   (-0.05, 0.11) | 0.13   (0.04, 0.22) | 0.08   (-0.02, 0.18) | 0.24   (0.09, 0.39) | 2.61E-03 | 2.32E-01 |  |
| **Hemoglobin and Porphyrin** |  |  |  |  |  |  |  |  |
| Bilirubin (E,Z or Z,E)* | -0.06   (-0.12, 0.01) | 0.01   (-0.06, 0.08) | -0.01   (-0.09, 0.07) | 0.06   (-0.02, 0.14) | 0.20   (0.07, 0.32) | 4.07E-03 | 4.11E-01 |  |
| **Histidine** |  |  |  |  |  |  |  |  |
| 1-Methyl-5-Imidazolelactate | -0.19   (-0.31, -0.07) | -0.10   (-0.23, 0.03) | -0.06   (-0.21, 0.09) | 0.06   (-0.10, 0.21) | 0.25   (0.01, 0.48) | 6.61E-03 | 7.53E-01 |  |
| Cis-Urocanate | -0.09   (-0.16, -0.02) | -0.03   (-0.10, 0.05) | 0.04   (-0.05, 0.13) | 0.10   (0.01, 0.19) | 0.28   (0.14, 0.43) | 8.77E-05 | 2.19E-03 |  |
| Imidazole Lactate | -0.09   (-0.14, -0.04) | -0.05   (-0.10, 0.01) | -0.01   (-0.07, 0.05) | 0.03   (-0.04, 0.10) | 0.12   (0.02, 0.23) | 2.45E-03 | 2.13E-01 |  |
| **Inositol** |  |  |  |  |  |  |  |  |
| Myo-Inositol | -0.08   (-0.14, -0.03) | -0.03   (-0.08, 0.03) | -0.07   (-0.13, -0.01) | 0.03   (-0.03, 0.10) | 0.11   (0.01, 0.21) | 2.21E-03 | 1.74E-01 |  |
| **Leucine, Isoleucine and Valine** |  |  |  |  |  |  |  |  |
| 2-Hydroxy-3-Methylvalerate | -0.14   (-0.22, -0.07) | -0.06   (-0.14, 0.02) | 0.04   (-0.06, 0.13) | 0.15   (0.06, 0.25) | 0.24   (0.09, 0.38) | 7.57E-05 | 1.82E-03 |  |
| 3-Hydroxy-2-Ethylpropionate | -0.09   (-0.14, -0.04) | 0.02   (-0.03, 0.07) | 0.04   (-0.03, 0.10) | 0.10   (0.04, 0.16) | 0.11   (0.01, 0.20) | 5.06E-03 | 5.26E-01 |  |
| 3-Methyl-2-Oxobutyrate | -0.06   (-0.10, -0.03) | -0.01   (-0.05, 0.03) | 0.04   (-0.00, 0.09) | 0.02   (-0.02, 0.07) | 0.09   (0.02, 0.16) | 5.43E-03 | 5.92E-01 |  |
| 3-Methyl-2-Oxovalerate | -0.08   (-0.12, -0.04) | -0.01   (-0.05, 0.03) | 0.06   (0.01, 0.11) | 0.03   (-0.02, 0.08) | 0.13   (0.05, 0.21) | 1.02E-03 | 5.49E-02 |  |
| 3-Methylglutarylcarnitine (2) | 0.05   (-0.05, 0.15) | 0.12   (0.02, 0.23) | 0.07   (-0.05, 0.19) | -0.02   (-0.15, 0.11) | -0.29   (-0.49, -0.09) | 3.76E-03 | 3.76E-01 |  |
| 4-Methyl-2-Oxopentanoate | -0.10   (-0.14, -0.05) | -0.03   (-0.07, 0.02) | 0.04   (-0.01, 0.10) | 0.05   (-0.01, 0.10) | 0.14   (0.06, 0.22) | 1.26E-04 | 3.79E-03 |  |
| Alpha-Hydroxyisocaproate | -0.08   (-0.13, -0.03) | -0.03   (-0.09, 0.02) | 0.04   (-0.03, 0.10) | 0.02   (-0.05, 0.08) | 0.14   (0.04, 0.24) | 5.69E-03 | 6.37E-01 |  |
| Alpha-Hydroxyisovalerate | -0.13   (-0.20, -0.06) | -0.08   (-0.15, -0.01) | 0.01   (-0.07, 0.09) | 0.21   (0.13, 0.29) | 0.41   (0.28, 0.53) | 3.77E-14 | 2.26E-13 |  |
| Tiglylcarnitine (C5:1-Dc) | -0.11   (-0.17, -0.05) | -0.03   (-0.10, 0.03) | 0.02   (-0.06, 0.09) | 0.13   (0.05, 0.21) | 0.19   (0.08, 0.31) | 6.02E-05 | 1.14E-03 |  |
| **Lysophospholipid** |  |  |  |  |  |  |  |  |
| 1-Palmitoyl-GPG (16:0)* | -0.10   (-0.16, -0.03) | -0.04   (-0.11, 0.02) | -0.04   (-0.12, 0.03) | -0.00   (-0.08, 0.07) | 0.18   (0.06, 0.29) | 1.35E-03 | 8.97E-02 |  |
| **Partially Characterized Molecules** |  |  |  |  |  |  |  |  |
| Bilirubin Degradation Product, C17h18n2o4 (3)** | -0.12   (-0.20, -0.04) | -0.02   (-0.10, 0.07) | -0.03   (-0.12, 0.07) | 0.01   (-0.09, 0.11) | 0.19   (0.03, 0.34) | 8.86E-03 | 1.00E+00 |  |
| Branched-Chain, Straight-Chain, or Cyclopropyl 10:1 Fatty Acid (1)* | -0.18   (-0.27, -0.10) | -0.02   (-0.11, 0.07) | 0.08   (-0.03, 0.18) | 0.02   (-0.08, 0.13) | 0.25   (0.09, 0.41) | 1.25E-03 | 7.86E-02 |  |
| Pentose Acid* | -0.09   (-0.21, 0.03) | -0.11   (-0.23, 0.02) | 0.02   (-0.13, 0.16) | 0.44   (0.29, 0.58) | 0.51   (0.28, 0.74) | 3.14E-07 | 3.77E-06 |  |
| **Pentose** |  |  |  |  |  |  |  |  |
| Arabitol/Xylitol | -0.02   (-0.06, 0.02) | 0.00   (-0.04, 0.04) | 0.03   (-0.02, 0.08) | 0.09   (0.04, 0.14) | 0.14   (0.06, 0.21) | 1.31E-03 | 8.36E-02 |  |
| Arabonate/Xylonate | -0.03   (-0.08, 0.01) | -0.03   (-0.08, 0.02) | 0.02   (-0.04, 0.07) | 0.12   (0.06, 0.18) | 0.13   (0.04, 0.23) | 7.87E-04 | 4.01E-02 |  |
| **Phenylalanine** |  |  |  |  |  |  |  |  |
| N-Acetylphenylalanine | -0.04   (-0.09, 0.00) | 0   (-0.04, 0.05) | 0.05   (-0.01, 0.10) | 0.10   (0.05, 0.16) | 0.16   (0.08, 0.25) | 3.15E-04 | 1.28E-02 |  |
| **Polyamine** |  |  |  |  |  |  |  |  |
| N-Acetylputrescine | -0.03   (-0.08, 0.01) | -0.02   (-0.07, 0.02) | 0.03   (-0.03, 0.08) | 0.06   (0.01, 0.12) | 0.14   (0.05, 0.22) | 1.98E-03 | 1.52E-01 |  |
| **Pregnenolone Steroids** |  |  |  |  |  |  |  |  |
| Pregnenediol Disulfate (C21H34O8S2)* | -0.22   (-0.32, -0.11) | -0.06   (-0.17, 0.05) | -0.09   (-0.22, 0.04) | 0.12   (-0.01, 0.25) | 0.29   (0.09, 0.49) | 1.19E-04 | 3.45E-03 |  |
| Purine, Adenine containing |  |  |  |  |  |  |  |  |
| N6-Succinyladenosine | -0.01   (-0.05, 0.02) | -0.00   (-0.04, 0.03) | -0.03   (-0.07, 0.01) | 0.03   (-0.02, 0.07) | 0.12   (0.05, 0.18) | 2.21E-03 | 1.78E-01 |  |
| Purine, Guanine containing |  |  |  |  |  |  |  |  |
| 7-Methylguanine | -0.02   (-0.05, 0.01) | -0.04   (-0.07, -0.01) | 0.02   (-0.01, 0.06) | 0.04   (0.00, 0.07) | 0.12   (0.07, 0.18) | 2.16E-05 | 3.89E-04 |  |
| **Pyrimidine, Uracil containing** |  |  |  |  |  |  |  |  |
| 3-Ureidopropionate | -0.04   (-0.08, 0.00) | -0.04   (-0.08, 0.00) | 0.02   (-0.03, 0.07) | 0.10   (0.05, 0.15) | 0.08   (0.00, 0.16) | 3.02E-03 | 2.95E-01 |  |
| **Tocopherol** |  |  |  |  |  |  |  |  |
| Gamma-CEHC | 0.10   (0.02, 0.18) | -0.02   (-0.11, 0.07) | -0.03   (-0.13, 0.07) | -0.12   (-0.22, -0.01) | -0.23   (-0.39, -0.07) | 3.71E-03 | 3.67E-01 |  |
| **Tryptophan** |  |  |  |  |  |  |  |  |
| 8-Methoxykynurenate | -0.11   (-0.20, -0.02) | 0.01   (-0.08, 0.09) | -0.02   (-0.12, 0.09) | 0.18   (0.07, 0.28) | 0.23   (0.06, 0.39) | 1.23E-03 | 7.33E-02 |  |
| N-Acetylkynurenine (2) | -0.13   (-0.22, -0.05) | -0.01   (-0.11, 0.08) | 0.04   (-0.07, 0.15) | 0.21   (0.10, 0.32) | 0.27   (0.10, 0.44) | 2.02E-04 | 7.69E-03 |  |
| N-Acetyltryptophan | -0.05   (-0.10, -0.01) | -0.01   (-0.05, 0.04) | 0.00   (-0.05, 0.06) | 0.11   (0.05, 0.16) | 0.13   (0.05, 0.22) | 2.83E-04 | 1.11E-02 |  |
| Indolelactate | -0.06   (-0.11, -0.01) | -0.04   (-0.09, 0.01) | 0.01   (-0.05, 0.06) | 0.14   (0.08, 0.20) | 0.13   (0.04, 0.23) | 1.56E-04 | 5.15E-03 |  |
| Kynurenate | -0.11   (-0.16, -0.05) | -0.00   (-0.06, 0.05) | 0.08   (0.01, 0.14) | 0.13   (0.07, 0.20) | 0.11   (0.00, 0.21) | 2.88E-03 | 2.68E-01 |  |
| Xanthurenate | -0.17   (-0.25, -0.08) | -0.08   (-0.16, 0.01) | 0.04   (-0.06, 0.14) | 0.21   (0.10, 0.31) | 0.17   (0.01, 0.33) | 4.04E-04 | 1.93E-02 |  |
| **Tyrosine** |  |  |  |  |  |  |  |  |
| 3-(4-Hydroxyphenyl)Lactate | -0.08   (-0.12, -0.03) | -0.08   (-0.13, -0.03) | 0.01   (-0.05, 0.06) | 0.10   (0.04, 0.16) | 0.11   (0.02, 0.21) | 1.61E-04 | 5.48E-03 |  |
| N-Acetyltyrosine | -0.06   (-0.11, -0.00) | -0.02   (-0.07, 0.04) | 0.06   (-0.00, 0.13) | 0.14   (0.07, 0.21) | 0.14   (0.03, 0.24) | 3.02E-03 | 2.93E-01 |  |
| **Urea cycle; Arginine and Proline** |  |  |  |  |  |  |  |  |
| N2,N5-Diacetylornithine | -0.12   (-0.20, -0.04) | -0.11   (-0.19, -0.03) | 0.02   (-0.08, 0.11) | 0.27   (0.17, 0.37) | 0.50   (0.35, 0.65) | 6.58E-14 | 5.27E-13 |  |
| **Xanthine** |  |  |  |  |  |  |  |  |
| 1,3,7-Trimethylurate | -0.52   (-0.69, -0.36) | -0.34   (-0.51, -0.17) | -0.09   (-0.30, 0.11) | 0.08   (-0.13, 0.29) | 0.31   (-0.02, 0.63) | 1.12E-04 | 3.03E-03 |  |
| 1,3-Dimethylurate | -0.24   (-0.34, -0.15) | -0.14   (-0.24, -0.04) | 0.03   (-0.08, 0.15) | 0.17   (0.05, 0.29) | 0.21   (0.02, 0.39) | 1.19E-04 | 3.40E-03 |  |
| 1,7-Dimethylurate | -0.46   (-0.59, -0.33) | -0.24   (-0.38, -0.11) | -0.18   (-0.34, -0.02) | 0.03   (-0.13, 0.20) | 0.00   (-0.25, 0.26) | 6.76E-03 | 7.84E-01 |  |
| 1-Methylurate | -0.22   (-0.33, -0.12) | -0.14   (-0.25, -0.03) | 0.03   (-0.09, 0.16) | 0.24   (0.11, 0.37) | 0.15   (-0.05, 0.35) | 1.35E-03 | 8.86E-02 |  |
| 1-Methylxanthine | -0.45   (-0.59, -0.32) | -0.24   (-0.38, -0.11) | -0.12   (-0.28, 0.04) | 0.12   (-0.04, 0.29) | 0.07   (-0.19, 0.33) | 1.57E-03 | 1.13E-01 |  |
| 3,7-Dimethylurate | 0.12   (0.03, 0.22) | 0.21   (0.10, 0.31) | 0.15   (0.04, 0.27) | 0.04   (-0.09, 0.16) | -0.17   (-0.36, 0.02) | 5.57E-03 | 6.18E-01 |  |
| Caffeine | -0.73   (-0.94, -0.51) | -0.47   (-0.70, -0.25) | -0.22   (-0.49, 0.04) | 0.08   (-0.19, 0.35) | 0.24   (-0.17, 0.66) | 3.29E-04 | 1.38E-02 |  |
| Paraxanthine | -0.50   (-0.65, -0.36) | -0.22   (-0.37, -0.07) | -0.08   (-0.26, 0.09) | 0.14   (-0.04, 0.32) | 0.18   (-0.10, 0.47) | 3.32E-04 | 1.44E-02 |  |
| Theophylline | -0.59   (-0.75, -0.43) | -0.37   (-0.54, -0.20) | -0.15   (-0.35, 0.04) | 0.09   (-0.12, 0.29) | 0.12   (-0.19, 0.44) | 4.04E-04 | 1.94E-02 |  |
| ^a^Metabolite and lipid species were log-transformed; Multivariable linear regression models were performed to calculate adjusted least square mean, and p for trend values are presented to access significance across categories.  ^b^89 out of 857 lipid species and 123 out of 828 metabolites were significant at the FDR p-value < 0.01 threshold and were included in the table.  ^c^Multiple hypothesis testing was corrected using the False Discovery Rate (FDR) method.  ^d^Multiple hypothesis testing was corrected using the Bonferroni method. | | | | | | | | |

**Supplementary Table S2. Adjusted Least Square Means of Lipid Species and Metabolites Associated with Frequency of Coffee Intake^ab^**

| **Metabolites** | **Never or <1 Serving** | **1 Serving** | **2-6 Servings** | **≥7 servings** | **FDR**  **P_trend_^c^** | **Bonferroni**  **P_trend_^d^** |  |
| --- | --- | --- | --- | --- | --- | --- | --- |
|  | **LSM**  **(95%CI)** | **LSM**  **(95%CI)** | **LSM**  **(95%CI)** | **LSM**  **(95%CI)** |  |  |  |
| **Benzoate** |  |  |  |  |  |  |  |
| 3-(3-Hydroxyphenyl)Propionate | -0.23   (-0.35, -0.10) | -0.19   (-0.52, 0.14) | -0.26   (-0.49, -0.03) | 0.23   (0.12, 0.34) | 4.13E-03 | 1.61E-01 |  |
| 3-Hydroxyhippurate | -0.38   (-0.53, -0.22) | -0.18   (-0.57, 0.22) | -0.20   (-0.47, 0.08) | 0.27   (0.14, 0.40) | 4.56E-04 | 1.64E-02 |  |
| 3-Methoxycatechol Sulfate (2) | -0.19   (-0.30, -0.09) | -0.21   (-0.50, 0.07) | -0.22   (-0.41, -0.02) | 0.14   (0.05, 0.24) | 9.82E-03 | 4.51E-01 |  |
| 3-Methyl Catechol Sulfate (1) | -0.93   (-1.10, -0.75) | -0.87   (-1.32, -0.42) | -0.09   (-0.40, 0.22) | 0.60   (0.45, 0.75) | 1.75E-23 | 8.75E-23 |  |
| 3-Methyl Catechol Sulfate (2) | -0.82   (-0.97, -0.67) | -0.53   (-0.91, -0.15) | -0.39   (-0.65, -0.13) | 0.12   (-0.00, 0.25) | 6.99E-10 | 1.19E-08 |  |
| 4-Ethylcatechol Sulfate | -0.69   (-0.85, -0.53) | -0.65   (-1.07, -0.23) | 0.03   (-0.26, 0.32) | 0.66   (0.52, 0.81) | 3.34E-21 | 2.00E-20 |  |
| 4-Vinylcatechol Sulfate | -0.76   (-0.93, -0.59) | -0.76   (-1.20, -0.32) | -0.31   (-0.61, -0.01) | 0.20   (0.06, 0.35) | 1.98E-10 | 3.17E-09 |  |
| Catechol Sulfate | -0.33   (-0.43, -0.23) | -0.34   (-0.59, -0.09) | -0.07   (-0.25, 0.10) | 0.27   (0.18, 0.35) | 6.35E-12 | 7.61E-11 |  |
| Guaiacol Sulfate | -0.38   (-0.49, -0.26) | -0.24   (-0.53, 0.06) | -0.03   (-0.24, 0.17) | 0.30   (0.20, 0.40) | 1.88E-09 | 3.57E-08 |  |
| O-Cresol Sulfate | -0.32   (-0.47, -0.16) | -0.29   (-0.69, 0.10) | 0.03   (-0.24, 0.30) | 0.49   (0.36, 0.62) | 1.07E-08 | 2.47E-07 |  |
| **Chemical** |  |  |  |  |  |  |  |
| 3-Acetylphenol Sulfate | -0.51   (-0.66, -0.37) | -0.43   (-0.81, -0.06) | -0.17   (-0.43, 0.09) | 0.30   (0.17, 0.43) | 3.92E-09 | 8.24E-08 |  |
| 3-Hydroxy-2-Methylpyridine Sulfate | -0.85   (-1.07, -0.62) | -0.66   (-1.25, -0.07) | -0.18   (-0.58, 0.23) | 0.56   (0.37, 0.76) | 6.89E-11 | 8.95E-10 |  |
| 3-Hydroxypyridine Sulfate | -1.24   (-1.44, -1.04) | -0.74   (-1.27, -0.21) | 0.12   (-0.25, 0.49) | 1.07   (0.89, 1.25) | 5.79E-32 | 1.16E-31 |  |
| 5-Hydroxy-2-Methylpyridine Sulfate | -0.70   (-0.91, -0.48) | -0.67   (-1.23, -0.11) | -0.73   (-1.11, -0.34) | 0.02   (-0.16, 0.21) | 6.47E-03 | 2.78E-01 |  |
| **Dipeptide** |  |  |  |  |  |  |  |
| Cyclo(Leu-Pro) | -0.20   (-0.29, -0.11) | -0.26   (-0.49, -0.03) | -0.23   (-0.39, -0.08) | 0.07   (-0.01, 0.15) | 4.82E-03 | 1.98E-01 |  |
| Cyclo(Pro-Val) | -0.15   (-0.23, -0.08) | -0.22   (-0.41, -0.03) | -0.02   (-0.15, 0.11) | 0.05   (-0.01, 0.12) | 6.94E-04 | 2.57E-02 |  |
| **Fatty Acid** |  |  |  |  |  |  |  |
| 2-Hydroxyglutarate | -0.14   (-0.19, -0.09) | -0.09   (-0.22, 0.03) | -0.02   (-0.10, 0.07) | 0.08   (0.04, 0.12) | 5.14E-06 | 1.64E-04 |  |
| 9,10-Dihome | 0.23   (0.13, 0.34) | 0.33   (0.06, 0.61) | -0.06   (-0.25, 0.13) | 0.03   (-0.07, 0.12) | 9.82E-03 | 4.52E-01 |  |
| Maleate | -0.14   (-0.23, -0.06) | -0.15   (-0.37, 0.07) | 0.05   (-0.10, 0.20) | 0.42   (0.35, 0.50) | 2.66E-13 | 2.93E-12 |  |
| **Food Component/Plant** |  |  |  |  |  |  |  |
| 3-Ethylcatechol Sulfate (1) | -0.53   (-0.65, -0.40) | -0.63   (-0.96, -0.30) | -0.19   (-0.42, 0.03) | 0.12   (0.01, 0.23) | 8.79E-10 | 1.58E-08 |  |
| 4-Acetylcatechol Sulfate (1) | -0.43   (-0.57, -0.29) | -0.39   (-0.76, -0.03) | -0.15   (-0.40, 0.10) | 0.27   (0.14, 0.39) | 2.11E-07 | 5.68E-06 |  |
| 4-Vinylguaiacol Sulfate | -0.75   (-0.92, -0.58) | -0.62   (-1.06, -0.18) | -0.42   (-0.73, -0.12) | 0.09   (-0.06, 0.24) | 1.02E-06 | 2.95E-05 |  |
| N-(2-Furoyl)Glycine | -0.53   (-0.75, -0.31) | -0.20   (-0.76, 0.35) | 0.29   (-0.09, 0.68) | 1.03   (0.85, 1.22) | 6.70E-14 | 6.70E-13 |  |
| Caffeic Acid Sulfate | -0.78   (-0.96, -0.59) | -0.63   (-1.11, -0.16) | -0.61   (-0.93, -0.28) | 0.13   (-0.02, 0.29) | 3.31E-06 | 9.92E-05 |  |
| Dihydrocaffeate Sulfate (2) | -0.27   (-0.41, -0.12) | -0.73   (-1.10, -0.36) | -0.21   (-0.46, 0.05) | 0.29   (0.16, 0.42) | 3.88E-08 | 9.70E-07 |  |
| Dihydroferulate | -0.16   (-0.27, -0.04) | -0.06   (-0.36, 0.23) | 0.00   (-0.20, 0.21) | 0.25   (0.15, 0.35) | 2.28E-03 | 8.65E-02 |  |
| Ferulic Acid 4-Sulfate | -0.70   (-0.86, -0.54) | -0.51   (-0.93, -0.10) | -0.58   (-0.87, -0.30) | 0.02   (-0.12, 0.16) | 1.64E-04 | 5.74E-03 |  |
| Levulinate (4-Oxovalerate) | -0.19   (-0.27, -0.11) | -0.11   (-0.31, 0.09) | 0.00   (-0.14, 0.14) | 0.17   (0.10, 0.24) | 5.28E-06 | 1.74E-04 |  |
| Quinate | -2.31   (-2.51, -2.12) | -1.62   (-2.11, -1.12) | -0.54   (-0.88, -0.19) | 0.70   (0.53, 0.86) | 5.34E-56 | 5.34E-56 |  |
| **Nicotinate and Nicotinamide** |  |  |  |  |  |  |  |
| Trigonelline (N'-Methylnicotinate) | -0.95   (-1.08, -0.82) | -0.61   (-0.95, -0.27) | -0.29   (-0.53, -0.06) | 0.58   (0.46, 0.69) | 1.14E-31 | 3.43E-31 |  |
| **Phospholipid** |  |  |  |  |  |  |  |
| Choline | 0.02   (-0.01, 0.04) | 0.07   (0.01, 0.12) | -0.03   (-0.06, 0.01) | -0.02   (-0.04, 0.00) | 4.47E-03 | 1.79E-01 |  |
| **Polyamine** |  |  |  |  |  |  |  |
| 4-Acetamidobutanoate | 0.08   (0.04, 0.12) | 0.09   (-0.02, 0.19) | -0.03   (-0.10, 0.04) | -0.08   (-0.11, -0.04) | 8.68E-06 | 2.95E-04 |  |
| **TCA Cycle** |  |  |  |  |  |  |  |
| Citraconate/Glutaconate | -0.49   (-0.66, -0.33) | -0.25   (-0.66, 0.17) | 0.30   (0.02, 0.59) | 1.07   (0.93, 1.21) | 1.25E-24 | 4.99E-24 |  |
| **Tyrosine** |  |  |  |  |  |  |  |
| Gentisate | -0.23   (-0.33, -0.13) | -0.22   (-0.48, 0.04) | -0.12   (-0.30, 0.06) | 0.23   (0.14, 0.31) | 3.49E-06 | 1.08E-04 |  |
| Vanillylmandelate (Vma) | 0.08   (0.03, 0.12) | 0.11   (0.00, 0.23) | 0.02   (-0.06, 0.10) | -0.03   (-0.07, 0.01) | 6.29E-03 | 2.64E-01 |  |
| **Vitamin A** |  |  |  |  |  |  |  |
| Carotene Diol (2) | -0.07   (-0.13, -0.01) | -0.17   (-0.33, -0.02) | -0.02   (-0.13, 0.09) | 0.05   (0.00, 0.11) | 9.82E-03 | 4.49E-01 |  |
| **Xanthine** |  |  |  |  |  |  |  |
| 1,3,7-Trimethylurate | -0.84   (-0.99, -0.69) | -0.31   (-0.69, 0.08) | -0.42   (-0.69, -0.16) | 0.16   (0.03, 0.29) | 3.34E-08 | 8.02E-07 |  |
| 1,3-Dimethylurate | -0.43   (-0.51, -0.34) | -0.05   (-0.27, 0.17) | -0.27   (-0.43, -0.12) | 0.18   (0.11, 0.26) | 1.30E-07 | 3.39E-06 |  |
| 1,7-Dimethylurate | -0.70   (-0.82, -0.58) | -0.43   (-0.74, -0.12) | -0.32   (-0.53, -0.11) | 0.05   (-0.05, 0.16) | 1.97E-09 | 3.95E-08 |  |
| 1-Methylurate | -0.44   (-0.53, -0.34) | -0.19   (-0.43, 0.05) | -0.25   (-0.41, -0.08) | 0.24   (0.16, 0.32) | 1.05E-10 | 1.58E-09 |  |
| 1-Methylxanthine | -0.68   (-0.80, -0.56) | -0.50   (-0.81, -0.19) | -0.37   (-0.59, -0.16) | 0.11   (0.01, 0.22) | 8.39E-11 | 1.17E-09 |  |
| 5-Acetylamino-6-Amino-3-Methyluracil | -0.81   (-0.94, -0.68) | -0.41   (-0.74, -0.08) | -0.39   (-0.62, -0.16) | 0.25   (0.14, 0.36) | 2.88E-14 | 2.59E-13 |  |
| 5-Acetylamino-6-Formylamino-3-Methyluracil | -0.48   (-0.63, -0.33) | 0.01   (-0.38, 0.39) | -0.12   (-0.39, 0.14) | 0.42   (0.29, 0.55) | 9.21E-07 | 2.58E-05 |  |
| Caffeine | -1.11   (-1.30, -0.91) | -0.67   (-1.17, -0.16) | -0.63   (-0.98, -0.29) | 0.14   (-0.03, 0.31) | 6.14E-09 | 1.35E-07 |  |
| Paraxanthine | -0.78   (-0.91, -0.65) | -0.67   (-1.00, -0.34) | -0.39   (-0.62, -0.16) | 0.20   (0.09, 0.32) | 4.17E-16 | 3.33E-15 |  |
| Theophylline | -1.01   (-1.15, -0.87) | -0.62   (-0.99, -0.26) | -0.32   (-0.57, -0.07) | 0.16   (0.04, 0.29) | 1.51E-16 | 1.06E-15 |  |
| ^a^Metabolite and lipid species were log-transformed; Multivariable linear regression models were performed to calculate adjusted least square mean, and p for trend values are presented to access significance across categories.  ^b^46 out of 828 metabolites were significant at the FDR p-value < 0.01 threshold and were included in the table.  ^c^Multiple hypothesis testing was corrected using the False Discovery Rate (FDR) method.  ^d^Multiple hypothesis testing was corrected using the Bonferroni method. | | | | | | | |

**Supplementary Table S3. Adjusted Least Square Means of Lipid Species and Metabolite Associated with** **Frequency of Multivitamin Use^ab^**

| **Metabolite** | **Never** | **≤3 Days** | **4-6 Days** | **Everyday** | **FDR**  **P_trend_^c^** | **Bonferroni**  **P_trend_^d^** |
| --- | --- | --- | --- | --- | --- | --- |
|  | **LSM**  **(95%CI)** | **LSM**  **(95%CI)** | **LSM**  **(95%CI)** | **LSM**  **(95%CI)** |  |  |
| **Ascorbate and Aldarate** |  |  |  |  |  |  |
| Ascorbic Acid 3-Sulfate* | -0.14   (-0.20, -0.07) | 0.07   (-0.05, 0.19) | -0.06   (-0.21, 0.09) | 0.18   (0.10, 0.27) | 4.63E-03 | 7.41E-02 |
| Oxalate (Ethanedioate) | -0.19   (-0.24, -0.14) | -0.08   (-0.16, 0.00) | -0.10   (-0.21, 0.00) | 0.02   (-0.04, 0.08) | 2.40E-03 | 3.12E-02 |
| Threonate | -0.22   (-0.28, -0.17) | -0.08   (-0.17, 0.01) | -0.09   (-0.20, 0.02) | 0.05   (-0.02, 0.11) | 2.05E-05 | 2.05E-04 |
| **Dipeptide** |  |  |  |  |  |  |
| Cyclo(Leu-Pro) | -0.02   (-0.10, 0.06) | 0.02   (-0.12, 0.15) | -0.22   (-0.38, -0.05) | -0.20   (-0.30, -0.11) | 7.69E-03 | 1.31E-01 |
| **Fatty Acid, Dicarboxylate** |  |  |  |  |  |  |
| Hydroxy-Cmpf* | -0.22   (-0.35, -0.09) | -0.17   (-0.40, 0.06) | -0.09   (-0.38, 0.20) | 0.24   (0.07, 0.40) | 9.14E-03 | 1.64E-01 |
| **Food Component/Plant** |  |  |  |  |  |  |
| N-Oxalyl Glycine (Nog) | -0.09   (-0.14, -0.05) | 0.04   (-0.04, 0.12) | 0.03   (-0.07, 0.13) | 0.10   (0.04, 0.16) | 2.78E-03 | 3.89E-02 |
| Tartronate (Hydroxymalonate) | -0.25   (-0.32, -0.19) | -0.13   (-0.23, -0.02) | -0.10   (-0.23, 0.04) | 0.02   (-0.05, 0.10) | 1.23E-04 | 1.48E-03 |
| **Glycolysis, Gluconeogenesis, and Pyruvate** |  |  |  |  |  |  |
| Glycerate | -0.15   (-0.19, -0.11) | -0.06   (-0.13, 0.01) | -0.07   (-0.16, 0.01) | 0.07   (0.01, 0.12) | 2.16E-05 | 2.37E-04 |
| **Methionine, Cysteine, SAM and Taurine** |  |  |  |  |  |  |
| 2-Hydroxy-4-(Methylthio)Butanoic Acid | 0.10   (0.05, 0.16) | -0.03   (-0.12, 0.07) | -0.16   (-0.28, -0.04) | -0.31   (-0.38, -0.24) | 2.52E-14 | 7.55E-14 |
| **Nicotinate and Nicotinamide** |  |  |  |  |  |  |
| N1-Methyl-2-Pyridone-5-Carboxamide | -0.13   (-0.19, -0.07) | 0.03   (-0.07, 0.12) | -0.00   (-0.13, 0.12) | 0.21   (0.14, 0.28) | 1.24E-06 | 7.43E-06 |
| N1-Methyl-4-Pyridone-3-Carboxamide | -0.12   (-0.18, -0.06) | 0.03   (-0.08, 0.14) | -0.02   (-0.15, 0.12) | 0.23   (0.15, 0.31) | 9.10E-06 | 8.19E-05 |
| **Pantothenate and CoA** |  |  |  |  |  |  |
| Pantothenate | -0.12   (-0.17, -0.06) | 0.04   (-0.06, 0.13) | 0.20   (0.08, 0.31) | 0.47   (0.40, 0.54) | 1.56E-28 | 1.56E-28 |
| **Tocopherol** |  |  |  |  |  |  |
| Alpha-CEHC Sulfate | -0.04   (-0.12, 0.05) | 0.12   (-0.03, 0.27) | 0.28   (0.10, 0.47) | 0.52   (0.41, 0.62) | 6.80E-11 | 2.72E-10 |
| Alpha-Tocopherol | -0.06   (-0.09, -0.03) | -0.04   (-0.10, 0.01) | 0.07   (0.00, 0.13) | 0.05   (0.02, 0.09) | 2.68E-06 | 2.06E-05 |
| Gamma-Tocopherol/Beta-Tocopherol | 0.05   (-0.01, 0.10) | -0.03   (-0.12, 0.07) | -0.08   (-0.20, 0.04) | -0.24   (-0.31, -0.17) | 2.68E-06 | 2.14E-05 |
| **Tyrosine** |  |  |  |  |  |  |
| 3-(4-Hydroxyphenyl)Lactate | 0.03   (-0.01, 0.07) | -0.06   (-0.12, 0.01) | -0.08   (-0.16, 0.00) | -0.11   (-0.16, -0.06) | 2.98E-03 | 4.47E-02 |
| **Vitamin B6** |  |  |  |  |  |  |
| Pyridoxal | -0.02   (-0.07, 0.03) | 0.11   (0.02, 0.20) | 0.06   (-0.05, 0.17) | 0.31   (0.25, 0.38) | 1.82E-07 | 9.10E-07 |
| Pyridoxate | -0.19   (-0.29, -0.08) | 0.24   (0.06, 0.42) | 0.23   (0.00, 0.45) | 0.89   (0.76, 1.02) | 1.48E-21 | 2.97E-21 |
| ^a^Metabolite and lipid species were log-transformed; Multivariable linear regression models were performed to calculate adjusted least square mean, and p for trend values are presented to access significance across categories.  ^b^18 out of 828 metabolites were significant at the FDR p-value < 0.01 threshold and were included in the table.  ^c^Multiple hypothesis testing was corrected using the False Discovery Rate (FDR) method.  ^d^Multiple hypothesis testing was corrected using the Bonferroni method. | | | | | | |

**Supplementary Table S4. Adjusted Least Square Means of Lipid Species and Metabolites Associated with Frequency of Statin Use^ab^**

| **Lipid Species** | **Never** | **1-3 Days** | **≥4 Days** | **FDR**  **P_trend_^c^** | **Bonferroni**  **P_trend_^d^** |
| --- | --- | --- | --- | --- | --- |
|  | **LSM**  **(95%CI)** | **LSM**  **(95%CI)** | **LSM**  **(95%CI)** |  |  |
| **Cholesteryl Esters** |  |  |  |  |  |
| CE(16:0) | 5.81   (5.79, 5.83) | 5.77   (5.67, 5.88) | 5.67   (5.60, 5.74) | 9.35E-03 | 2.84E-01 |
| CE(17:0) | 1.91   (1.88, 1.93) | 1.95   (1.81, 2.10) | 1.73   (1.63, 1.82) | 9.35E-03 | 3.09E-01 |
| CE(18:0) | 3.74   (3.72, 3.76) | 3.70   (3.58, 3.81) | 3.56   (3.49, 3.64) | 1.54E-03 | 2.77E-02 |
| CE(18:1) | 6.13   (6.11, 6.14) | 6.10   (6.00, 6.20) | 5.98   (5.92, 6.05) | 6.35E-03 | 1.59E-01 |
| CE(18:3) | 3.90   (3.87, 3.93) | 3.98   (3.81, 4.14) | 3.66   (3.55, 3.77) | 1.00E-03 | 1.40E-02 |
| CE(20:1) | 0.11   (0.09, 0.13) | -0.04   (-0.14, 0.06) | -0.09   (-0.16, -0.03) | 7.80E-05 | 5.46E-04 |
| CE(20:2) | 0.65   (0.63, 0.67) | 0.60   (0.48, 0.72) | 0.48   (0.40, 0.56) | 8.68E-03 | 2.34E-01 |
| CE(22:4) | -0.70   (-0.73, -0.68) | -0.71   (-0.85, -0.58) | -0.92   (-1.01, -0.83) | 8.31E-04 | 9.03E-03 |
| CE(24:1) | -2.62   (-2.64, -2.60) | -2.77   (-2.88, -2.66) | -2.86   (-2.94, -2.79) | 2.93E-05 | 5.13E-05 |
| **Ceramides** |  |  |  |  |  |
| CER(14:0) | -3.97   (-3.99, -3.94) | -4.02   (-4.16, -3.88) | -4.17   (-4.26, -4.08) | 3.80E-03 | 8.37E-02 |
| CER(18:1) | -4.67   (-4.70, -4.64) | -4.49   (-4.66, -4.32) | -4.86   (-4.98, -4.75) | 9.35E-03 | 3.00E-01 |
| CER(22:1) | -2.96   (-2.99, -2.94) | -2.85   (-3.01, -2.69) | -3.15   (-3.26, -3.04) | 9.75E-03 | 3.41E-01 |
| CER(24:1) | 0.07   (0.05, 0.09) | 0.13   (-0.00, 0.25) | -0.08   (-0.17, 0.00) | 9.35E-03 | 3.00E-01 |
| CER(26:0) | -3.13   (-3.15, -3.11) | -3.21   (-3.34, -3.08) | -3.34   (-3.43, -3.25) | 1.54E-03 | 2.67E-02 |
| **Hexosylceramides** |  |  |  |  |  |
| HCER(14:0) | -4.07   (-4.09, -4.04) | -4.07   (-4.21, -3.93) | -4.28   (-4.38, -4.19) | 1.12E-03 | 1.68E-02 |
| HCER(16:0) | -0.04   (-0.06, -0.02) | -0.16   (-0.29, -0.04) | -0.29   (-0.37, -0.21) | 2.93E-05 | 1.17E-04 |
| HCER(18:0) | -2.21   (-2.23, -2.18) | -2.29   (-2.42, -2.15) | -2.45   (-2.54, -2.36) | 3.47E-04 | 3.12E-03 |
| HCER(18:1) | -4.02   (-4.05, -4.00) | -4.02   (-4.16, -3.89) | -4.23   (-4.31, -4.14) | 1.14E-03 | 1.83E-02 |
| HCER(20:0) | -2.29   (-2.31, -2.26) | -2.39   (-2.52, -2.25) | -2.56   (-2.65, -2.47) | 2.93E-05 | 7.04E-05 |
| HCER(22:0) | -0.12   (-0.15, -0.10) | -0.21   (-0.35, -0.08) | -0.39   (-0.48, -0.30) | 3.95E-05 | 2.37E-04 |
| HCER(22:1) | -2.92   (-2.94, -2.89) | -2.93   (-3.04, -2.81) | -3.10   (-3.18, -3.02) | 8.31E-04 | 9.22E-03 |
| HCER(24:0) | 0.11   (0.09, 0.13) | 0.01   (-0.12, 0.14) | -0.15   (-0.23, -0.06) | 3.51E-05 | 1.75E-04 |
| HCER(24:1) | -0.28   (-0.30, -0.25) | -0.43   (-0.58, -0.29) | -0.58   (-0.68, -0.48) | 2.93E-05 | 1.17E-04 |
| HCER(26:1) | -4.62   (-4.64, -4.60) | -4.65   (-4.77, -4.54) | -4.77   (-4.85, -4.70) | 9.53E-03 | 3.24E-01 |
| **Lactosylceramides** |  |  |  |  |  |
| LCER(14:0) | -2.36   (-2.39, -2.33) | -2.49   (-2.65, -2.33) | -2.63   (-2.73, -2.52) | 8.31E-04 | 9.97E-03 |
| **Phosphatidylcholines** |  |  |  |  |  |
| PC(16:0/18:2) | 6.43   (6.41, 6.45) | 6.32   (6.21, 6.43) | 6.27   (6.19, 6.34) | 6.35E-03 | 1.57E-01 |
| PC(18:0/18:2) | 5.43   (5.41, 5.45) | 5.34   (5.23, 5.45) | 5.26   (5.18, 5.33) | 1.72E-03 | 3.27E-02 |
| PC(18:2/16:1) | 2.54   (2.51, 2.56) | 2.48   (2.34, 2.61) | 2.35   (2.26, 2.44) | 9.35E-03 | 2.80E-01 |
| PC(18:2/20:2) | -0.33   (-0.35, -0.30) | -0.55   (-0.68, -0.42) | -0.56   (-0.64, -0.47) | 2.93E-03 | 6.15E-02 |
| **Phosphatidylinositols** |  |  |  |  |  |
| PI(16:0/18:2) | -1.04   (-1.07, -1.01) | -1.07   (-1.24, -0.91) | -1.28   (-1.39, -1.17) | 4.50E-03 | 1.12E-01 |
| PI(18:0/18:2) | 0.17   (0.14, 0.20) | 0.03   (-0.14, 0.20) | -0.15   (-0.27, -0.03) | 4.60E-05 | 3.68E-04 |
| PI(18:1/20:4) | -1.20   (-1.22, -1.18) | -1.23   (-1.37, -1.10) | -1.40   (-1.49, -1.31) | 2.23E-03 | 4.90E-02 |
| **Sphingomyelins** |  |  |  |  |  |
| SM(14:0) | 2.70   (2.67, 2.72) | 2.66   (2.52, 2.79) | 2.52   (2.43, 2.61) | 9.35E-03 | 2.73E-01 |
| SM(16:0) | 5.22   (5.20, 5.23) | 5.08   (5.00, 5.17) | 5.06   (5.00, 5.12) | 9.19E-04 | 1.19E-02 |
| SM(24:1) | 4.35   (4.33, 4.36) | 4.24   (4.15, 4.33) | 4.21   (4.16, 4.27) | 8.22E-03 | 2.14E-01 |
| **Metabolite** |  |  |  |  |  |
| **Drug – Analgesics, Anesthetics** |  |  |  |  |  |
| Salicyluric Glucuronide* | 0.24   (0.13, 0.35) | 0.62   (0.03, 1.21) | 1.18   (0.78, 1.57) | 7.58E-03 | 3.03E-02 |
| **Glycolysis, Gluconeogenesis, and Pyruvate** |  |  |  |  |  |
| 1,5-Anhydroglucitol (1,5-AG) | -0.09   (-0.14, -0.04) | -0.41   (-0.68, -0.14) | -0.58   (-0.76, -0.41) | 1.30E-03 | 3.91E-03 |
| Glucose | 0.01   (-0.01, 0.02) | 0.05   (-0.04, 0.13) | 0.19   (0.13, 0.24) | 1.93E-05 | 1.93E-05 |
| **Sterol** |  |  |  |  |  |
| Cholesterol | -0.00   (-0.02, 0.02) | -0.10   (-0.20, -0.01) | -0.18   (-0.24, -0.12) | 8.53E-04 | 1.71E-03 |
| ^a^Metabolite and lipid species were log-transformed; Multivariable linear regression models were performed to calculate adjusted least square mean, and p for trend values are presented to access significance across categories.  ^b^35 out of 857 lipid species and 4 out of 828 metabolites were significant at the FDR p-value < 0.01 threshold and were included in the table.  ^c^Multiple hypothesis testing was corrected using the False Discovery Rate (FDR) method.  ^d^Multiple hypothesis testing was corrected using the Bonferroni method. | | | | | |

**Supplementary Table S5. Overlap in Lipid Species between Exposures, Significant at FDR < 0.05**

| **Frequency of Alcohol intake-Frequency of Statin use** | **Lipid** | **Frequency of Alcohol intake Estimate (SE)** | **Frequency of Statin use Estimate (SE)** |
| --- | --- | --- | --- |
|  | CE(18:3) | 0.64 (0.19) | -0.76 (0.17) |
|  | CER(24:1) | 0.55 (0.15) | -0.49 (0.13) |

**Supplementary Table S6. Overlap in Metabolites between Exposures, Significant at FDR < 0.05**

| **Frequency of Alcohol intake-Frequency of Coffee intake** | **Metabolite** | **Frequency of Alcohol intake Estimate (SE)** | **Frequency of Coffee intake Estimate (SE)** |
| --- | --- | --- | --- |
|  | **Benzoate** |  |  |
|  | 3-Methyl Catechol Sulfate (1) | 2.78 (0.81) | 6.84 (0.62) |
|  | O-Cresol Sulfate | 2.97 (0.66) | 3.52 (0.55) |
|  | **Dipeptide** |  |  |
|  | Cyclo(Leu-Pro) | 1.36 (0.37) | 1.17 (0.32) |
|  | **Fatty Acid** |  |  |
|  | 2-Hydroxyglutarate | 1.04 (0.20) | 0.90 (0.17) |
|  | Maleate | 1.55 (0.38) | 2.47 (0.31) |
|  | **Food Component/Plant** |  |  |
|  | Levulinate (4-Oxovalerate) | 1.29 (0.33) | 1.48 (0.28) |
|  | Quinate | 4.65 (1.09) | 12.43 (0.69) |
|  | **Xanthine** |  |  |
|  | 1-Methylurate | 1.62 (0.42) | 2.39 (0.33) |
|  | 1-Methylxanthine | 2.04 (0.53) | 3.12 (0.43) |
|  | 1,3-Dimethylurate | 1.77 (0.38) | 1.84 (0.31) |
|  | 1,3,7-Trimethylurate | 3.11 (0.67) | 3.35 (0.54) |
|  | 1,7-Dimethylurate | 1.74 (0.52) | 2.86 (0.43) |
|  | Caffeine | 3.73 (0.86) | 4.56 (0.70) |
|  | Paraxanthine | 2.52 (0.58) | 4.11 (0.46) |
|  | Theophylline | 2.75 (0.65) | 4.60 (0.51) |
| **Frequency of Alcohol intake-Frequency of Multivitamin use** | **Metabolite** | **Frequency of Alcohol intake Estimate (SE)** | **Frequency of Multivitamin use Estimate (SE)** |
|  | **Dipeptide** |  |  |
|  | Cyclo(Leu-Pro) | 1.36 (0.37) | -1.00 (0.26) |
|  | **Tyrosine** |  |  |
|  | 3-(4-Hydroxyphenyl)lactate | 0.85 (0.19) | -0.53 (0.13) |
| **Frequency of Coffee intake-Frequency of Multivitamin intake** | **Metabolite** | **Frequency of Coffee intake Estimate (SE)** | **Frequency of Multivitamin use Estimate (SE)** |
|  | **Dipeptide** |  |  |
|  | Cyclo(Leu-Pro) | 1.17 (0.32) | -1.00 (0.26) |
|  |  |  |  |

**Supplementary Figure S1. Flow Diagram of Participant Selection**


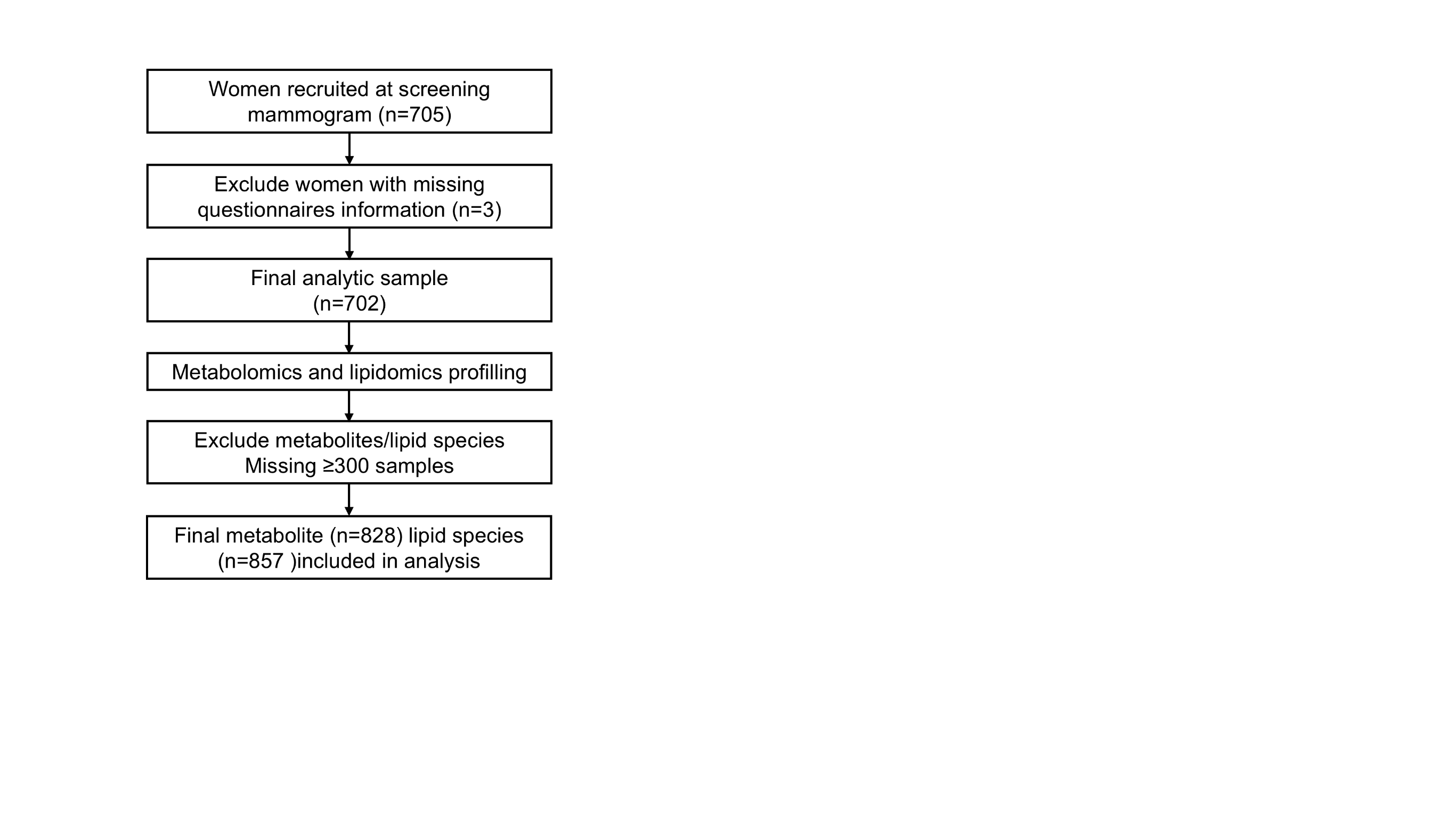


**Supplementary Figure S2. Heatmaps of Metabolites Associated with Frequency of Alcohol, Coffee, and Multivitamin**

**
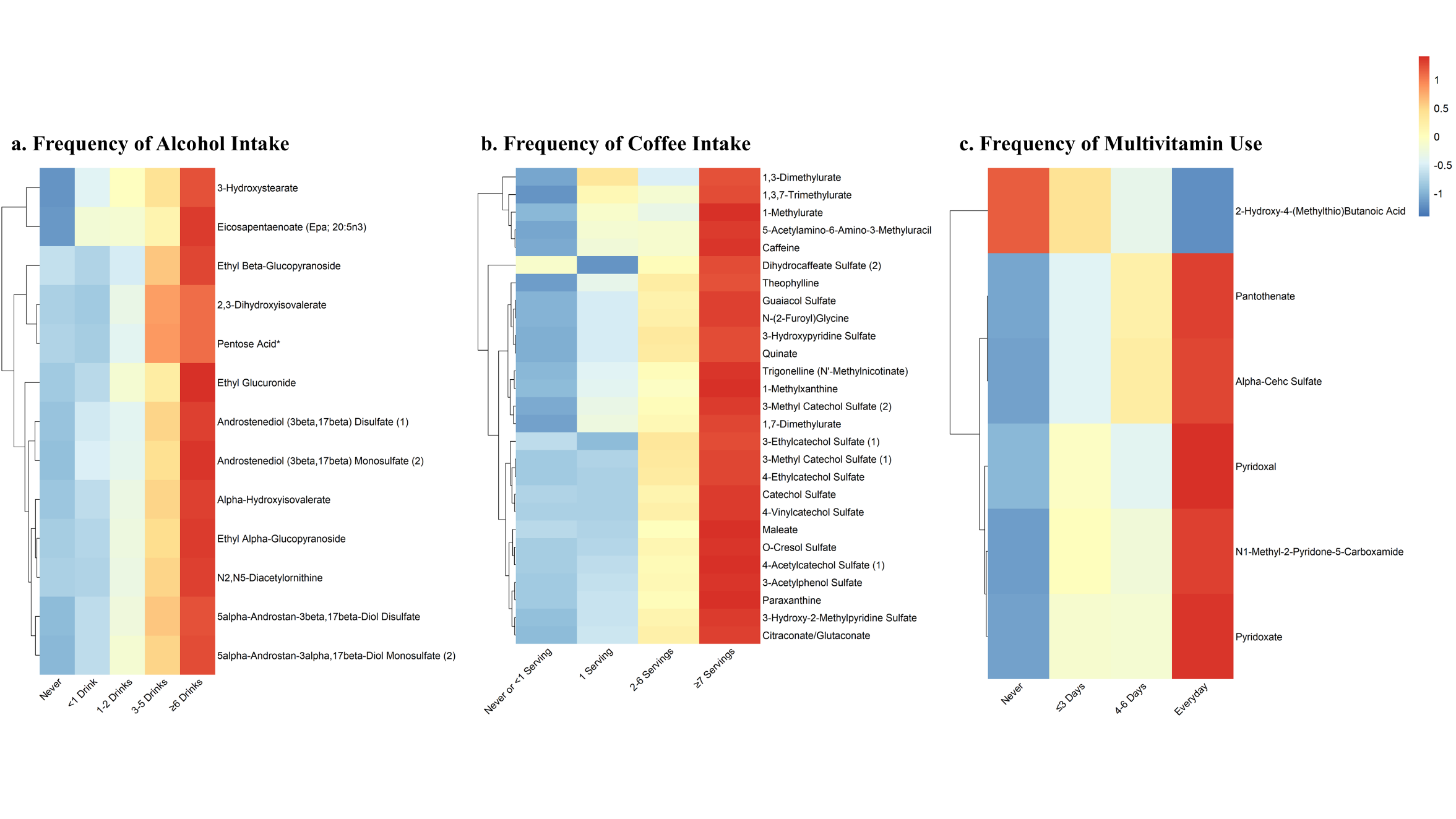
**

**Supplementary Figure S3. Sankey Plot of Significant Metabolite Subpathways across Exposure**

**
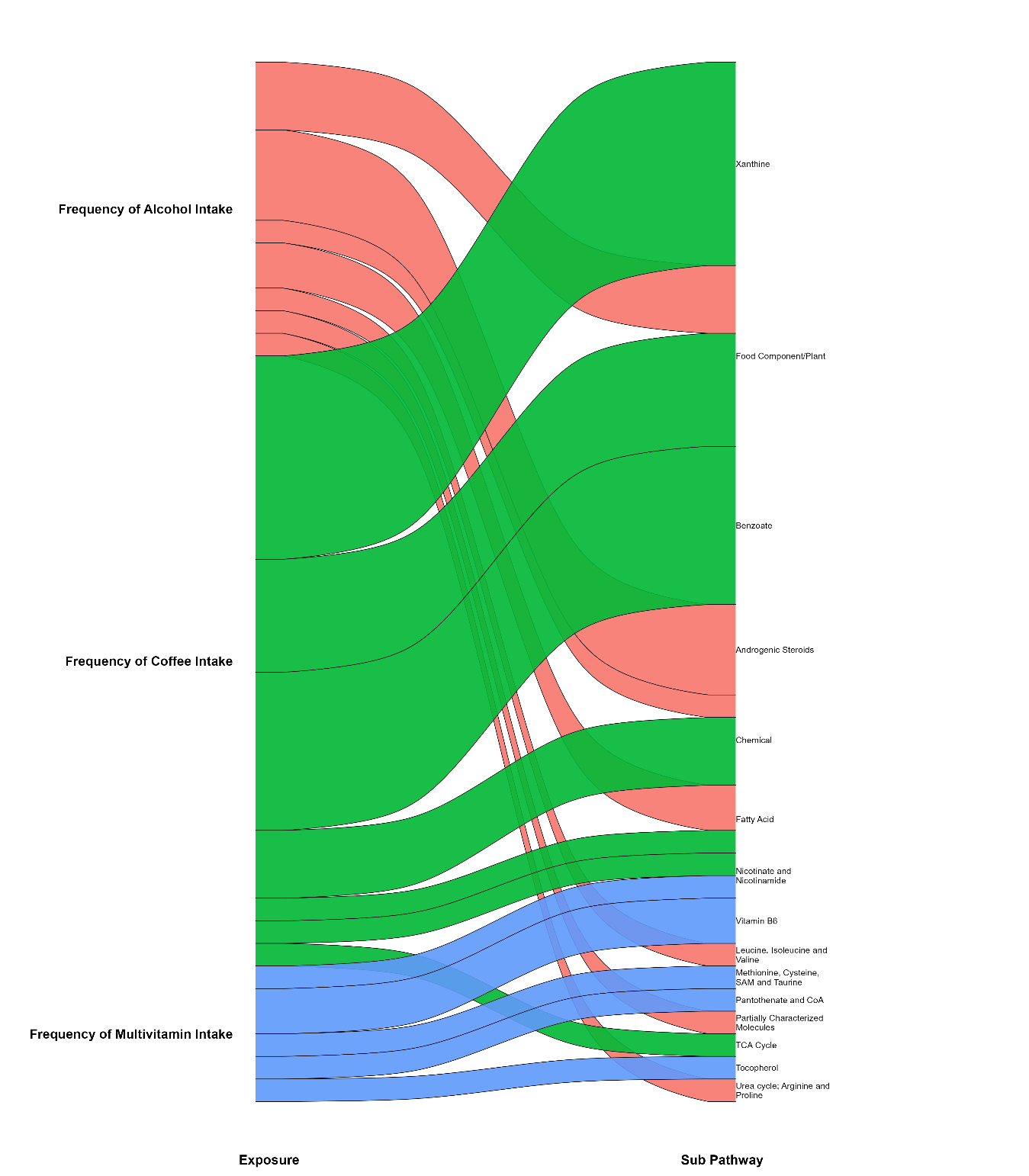
**

**Supplementary Figure S4. UpSet Plot of Significant Metabolite Overlap Across Exposures**

**
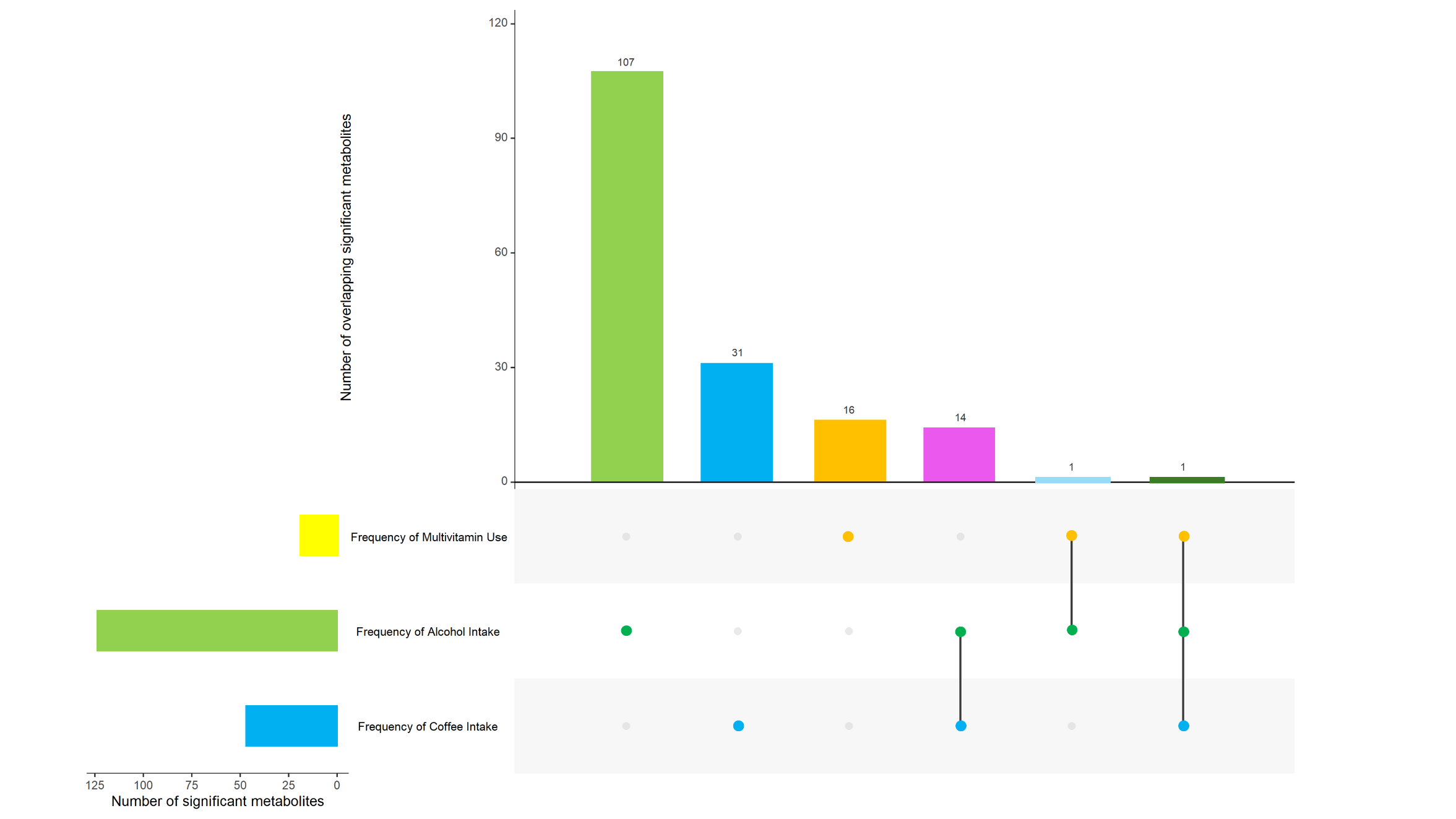
**
